# A high-throughput metabolomics in vitro platform for the characterization of hepatotoxicity

**DOI:** 10.1007/s10565-023-09809-6

**Published:** 2023-05-04

**Authors:** Sabina Ramirez-Hincapie, Barbara Birk, Philipp Ternes, Varun Giri, Volker Haake, Michael Herold, Franziska Maria Zickgraf, Andreas Verlohner, Hans-Albrecht Huener, Hennicke Kamp, Peter Driemert, Robert Landsiedel, Elke Richling, Dorothee Funk-Weyer, Bennard van Ravenzwaay

**Affiliations:** 1 BASF SE, Experimental Toxicology and Ecology, Ludwigshafen, Germany; 2 BASF Metabolome Solutions GmbH, Berlin, Germany; 3 Free University of Berlin, Pharmacy, Pharmacology and Toxicology, Berlin, Germany; 4 Food Chemistry and Toxicology, Department of Chemistry, University of Kaiserslautern-Landau, Kaiserslautern, Germany; 5 Environmental Sciences Consulting, Altrip, Germany

**Keywords:** Metabolomics, Toxicology in vitro, Toxicometabolomics, Mode of action, Liver toxicity, Hepatotoxicity, High throughput

## Abstract

Cell-based metabolomics provides multiparametric physiologically relevant readouts that can be highly advantageous for improved, biologically based decision making in early stages of compound development. Here, we present the development of a 96-well plate *LC*-*MS*/*MS-*based targeted metabolomics screening platform for the classification of liver toxicity modes of action (MoAs) in HepG2 cells. Different parameters of the workflow (cell seeding density, passage number, cytotoxicity testing, sample preparation, metabolite extraction, analytical method, and data processing) were optimized and standardized to increase the efficiency of the testing platform. The applicability of the system was tested with seven substances known to be representative of three different liver toxicity MoAs (peroxisome proliferation, liver enzyme induction, and liver enzyme inhibition). Five concentrations per substance, aimed at covering the complete dose-response curve, were analyzed and 221 uniquely identified metabolites were measured, annotated, and allocated in 12 different metabolite classes such as amino acids, carbohydrates, energy metabolism, nucleobases, vitamins and cofactors, and diverse lipid classes. Multivariate and univariate analyses showed a dose response of the metabolic effects, a clear differentiation between liver toxicity MoAs and resulted in the identification of metabolite patterns specific for each MoA. Key metabolites indicative of both general and mechanistic specific hepatotoxicity were identified. The method presented here offers a multiparametric, mechanistic-based, and cost-effective hepatotoxicity screening that provides MoA classification and sheds light into the pathways involved in the toxicological mechanism. This assay can be implemented as a reliable compound screening platform for improved safety assessment in early compound development pipelines.

**Graphical abstract:**

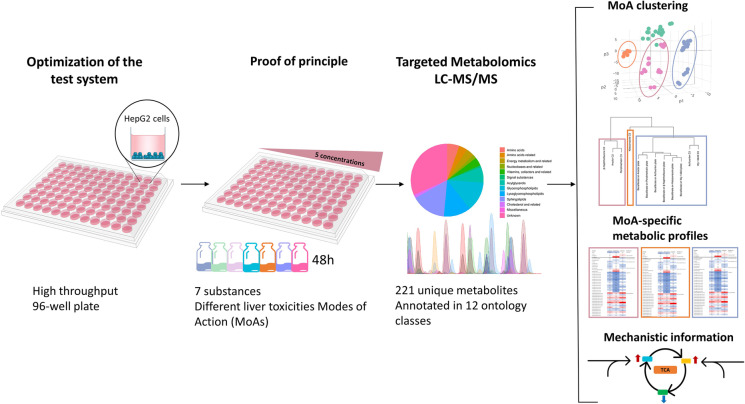

**Supplementary Information:**

The online version contains supplementary material available at 10.1007/s10565-023-09809-6.

## Introduction

Toxicological assessment is a critical step in chemical and drug development pipelines. The increasing number and complexity of candidate compounds keep challenging conventional toxicity evaluation procedures (Wang et al. 2020). In addition, the consecutive implementation of stringent regulatory frameworks (REACH EC No. 1907/2006, European Cosmetics Act EC No. 1223/2009, chemical strategy toward sustainability COM/2022) aiming to ensure human safety and environmental sustainability of both new and existing substances have resulted in increased testing needs (Crawford et al. 2017; van Dijk et al. 2021). Thus, to warrant the continued production and the development of new safe and sustainable chemicals, reliable, cost-effective, and high-throughput methods are needed.

The low throughput and high costs of traditional animal-based toxicity studies have rendered them impractical for assessing large numbers of compounds. In 2007, a new vision and roadmap for toxicity testing in the twenty-first century was proposed, consisting of moving away from utilizing large animal cohorts and observational sciences to incorporate more efficient and human relevant technologies that provide a better understanding of the mechanisms of toxicity (National Research Council, 2007).

The development and use of in vitro human cell-based assays has been a major step in the implementation of the Toxicology of the 21st Century (Tox21 program) and have been fundamental in the understanding of molecular mechanisms of toxicity and in the development of adverse outcome pathways (AOP) (Vinken 2013; Krewski et al. 2020). More recently, complex in vitro toxicity models (e.g., three-dimensional (3D) organoid models, hiPSCs derived systems, organ-on-a-chip platforms) addressing systemic toxicity endpoints have been developed (Plummer et al. 2019; Richards et al. 2020; Shinozawa et al. 2021). Despite the advantage they offer, the limited throughput, complexity, and high cost of these sophisticated in vitro models reduce their applicability. Additionally, toxicological in vitro testing has been based mainly on mono-parametric strategies (one question, one answer) which is time consuming and limits the full characterization of toxicological-related events (Dix et al. 2007).

The use of multiparametric “omics” technologies allows the simultaneous evaluation of multiple parameters in a single biological sample and offers a more comprehensive tool for elucidating the molecular and biochemical events underlaying organ toxicity (García-Cañaveras et al. 2016). In particular, metabolites represent the most downstream products and final outcome of expression of the genome, transcriptome and proteome, thus providing a snapshot of the biochemical and physiological status of a system including its response to external stressors (Guijas et al. 2018). For this reason, metabolomics is considered to be the omics technology which is closest to classical toxicology and has shown to have a similar sensitivity (Van Ravenzwaay et al. 2014). Metabolomics has been successfully implemented for more than a decade to identify toxicological mechanisms in rodent studies (Kamp et al. 2012; Van Ravenzwaay et al. 2014; Van Ravenzwaay et al. 2015). More recently, it has been used in combination with in vitro models to expand the investigation of organ toxicity (García-Cañaveras et al. 2016; Birk et al. 2021; Huang et al. 2021; Jeon et al. 2021).

The liver is one of the most frequent target organs of chemical toxicity. Drug-induced liver injury (DILI) represents the leading cause of failure in new pharmaceutical development and post approval compound withdrawals (Onakpoya et al. 2016). Therefore, hepatotoxicity is of primary concern in compound development and considerable efforts have been directed to the development of assays to evaluate liver toxicity (Mirahmad et al. 2022). Several liver cell lines have been successfully used for in vitro metabolomics to identify modes of action of liver toxicity (Cuykx et al. 2018b). However, in contrast to popular perception, so far, in vitro experiments and, in particular, metabolomics experiments are costly and material- and labor-intensive (García-Cañaveras et al. 2016; Cuykx et al. 2018b; Ramirez et al. 2018). We have previously developed a HepG2 cell-based metabolomics in vitro platform, capable of identifying and characterizing different modes of action (MoAs) of liver toxicity. Despite its good performance as a research tool, the assay was costly, complex, and time consuming (Ramirez et al. 2018). These factors have critically limited in vitro metabolomics throughput and scalability and prevented its implementation in high-throughput screening during early stages of compound development.

Improvements in the sensitivity of analytical techniques for metabolomics have opened the possibility of scaling the throughput in metabolomics (Dubuis et al. 2018; Zampieri et al. 2018; Anglada-Girotto et al. 2022; Malinowska et al. 2022). We have used this opportunity to develop and evaluate a highly standardized, 96-well in vitro metabolomics screening platform for the identification and classification of liver toxicity MoAs in HepG2 cells. Different parameters of the workflow such as cell seeding density, influence of passage number, cytotoxicity testing, sample preparation, metabolite extraction, analytical method, and data processing were optimized to perform with low biomass samples. This new methodology was then tested with seven compounds with known hepatotoxicity modes of action (MoA) in five different concentrations.

## Materials and methods

### Cell culture

HepG2 cells (ECACC, UK, maximum passage number 9) were maintained and grown on Dulbecco’s MEM media supplemented with 1% v/v of penicillin/streptomycin, l-glutamine (200 mM, 1% v/v), non-essential amino acids (100x, 1% v/v), and 10% FBS (PAN-Biotech, Aidenbach, Germany) in 75 cm^2^ culture flasks (TPP, Switzerland). For cell passaging (~ 80% confluency) media was removed and cells were washed twice with pre-warmed calcium and magnesium free Dulbecco’s PBS (PAN-Biotech, Aidenbach, Germany). Trypsin was used for cell detachment. Then, 20 mL culture medium was added, and single-cell suspensions were obtained by passing the suspension through a Combitip (Eppendorf, Germany). A fraction of the cell suspension was then transferred to a new culture vessel. For experiments, 15,000 cells per well (passage 5-9) were seeded in 96-well flat-bottom plates (TPP, Switzerland) and incubated for 24 h for cell attachment (37 °C and 5% CO^2^). Afterwards, culture media were exchanged, test substances applied, and plates incubated for 48 h (37 °C and 5% CO^2^).

### Test substances

Test substances (Table 1) were selected based on their known in vivo liver toxicity effects and different MoAs as well as results from previous in vitro studies in our lab (Ramirez et al. 2018). Acifluorfen, bezafibrate, wy-14643, β-naphthoflavone, pendimethalin, and ketoconazole were purchased from Sigma-Aldrich (Taufkirchen, Germany) and Aroclor 1254 from Chem-Service (West Chester, PA, USA). Purity of all substances ≥ 98%.

**Table 1:** Overview of test substances used for treatment of HepG2 cells for 48 h

Substance	CAS-Nr.	Chemical class	Category	MoA
Acifluorfen	50594-66-6	Diphenyl ether	Herbicide	Peroxisome proliferation
Bezafibrate	41859-67-0	Fibric acids	Hypolipidemic agents	Peroxisome proliferation
Wy-14643	50892-23-4	Pyrimidines	Hypolipidemic agents	Peroxisome proliferation
β-naphthoflavone	6051-87-2	Benzoflavone	Industrial chemical	Liver enzyme inducer
Aroclor 1254	11097-69-1	Polychlorinated biphenyl	Industrial chemical	Liver enzyme inducer
Pendimethalin	40487-42-1	Dinitroaniline	Herbicide	Liver enzyme inducer
Ketoconazole	65277-42-1	Imidazole derivative	Fungicide	Enzyme inhibitor

### Cytotoxicity and cell viability testing

Commercially available cytotoxicity (CellTox™ Green) and ATP content based (CellTiter-Glo®) assays (Promega GmbH, Walldorf, Germany) were multiplexed in a single 96-well plate following the manufacturer’s instructions. For positive controls, lysis solution 25X was added in wells containing vehicle control treated cells (0.5% DMSO). Then, 10X CellTox Green reagent was added in all wells, plates were shaken for 1 min, and incubated in the dark for 15 min at room temperature. Fluorescence was measured at λ_ex_ = 485–500 nm/λ_em_ = 520–530 nm in the GloMax®-Multi Detection System (Promega). Afterwards, wells were washed with PBS, 100 μL of Dulbecco’s MEM media were added to each well and subsequently 100 μL of 1X CellTiter-Glo were added. Plates were shaken for 2 min and incubated in the dark for 8 min at room temperature. Luminescence was measured in the GloMax®-Multi Detection System (Promega) and was normalized to the values of the vehicle control. Cytotoxicity and ATP cell viability analysis were carried out for range finder pre-tests and in parallel with metabolomics experiments in plates handled and treated exactly as the ones used for metabolite profiling.

### Range finder experiments for dose setting

In order to define appropriate dose levels, range finder experiments were performed prior to metabolome experiments. Substances were administered to HepG2 cells in increasing concentrations and incubated for 48 h (6 replicates per concentration). Viability and cytotoxicity tests were performed as described previously. Luminescence values resulting from CellTiter-Glo® assays were used to build dose response curves. Curve fitting and effective concentrations (ECs) values were calculated in R using three-parameter Weibull model (W1.3). To obtain a full metabolome-based dose-response effect, five concentrations (EC1, EC5, EC15, EC50, EC85) were selected for metabolome experiments (Table 2). EC1 and EC5 were selected to evaluate mild metabolic effects, EC15 was selected to obtain a robust substance-related effect; however, within a low cytotoxicity range and EC50 and EC85 were chosen to identify cytotoxic-related metabolite patterns. Calculated ECs values were rounded to the nearest integer number for dose selection.

**Table 2:** Concentrations selected to perform the metabolomics experiments based on range finder experiments

Substance	EC1 (μM)	EC5 (μM)	EC15 (μM)	EC50 (μM)	EC85 (μM)
Bezafibrate^a^			1000		
Acifluorfen	50	100	200	500	800
Wy-14643	25	50	150	500	1000
β-naphthoflavone	0.1	1	10	100	700
Aroclor 1254	21	38	56	94	133
Pendimethalin	24	39	56	94	157
Ketoconazole	5^b^	0.2	1	10	50

^a^Bezafibrate 1000 μM was used as positive control in each experiment

^b^For Ketoconazole, the EC1 was excluded and a concentration between the EC15 and EC50 was selected instead. ECs were estimated based on the ATP dose response curves generated in the range finder experiments

### Live-cell imaging

To monitor cell proliferation, total well confluence was obtained by real-time cell imaging analysis using IncuCyte S3 device placed in a normal incubator at 37 °C with 5% CO^2^. Whole-well scans were taken every 6 h during the duration of the assay and evaluated using automated phase-contrast analysis (phase mask).

### Metabolomics experiments

After 24 h of cell attachment, substances were administered in 0.5% DMSO to HepG2 cells in 5 concentrations (EC_1(ATP)_, EC_5(ATP)_, EC_15(ATP)_, EC_50(ATP)_, EC_85(ATP)_) and incubated for 48 h. For each substance, one 96-well plate was set up with 6 replicates per concentration, 12 replicates for vehicle controls (yielding a final concentration of 0.5% DMSO in the well), 6 replicates for positive controls (Bezafibrate 1000 μM), and 6 replicates for blank controls (media without cells). To minimize potential evaporation, the outer rows and columns of the plate were omitted and filled with PBS instead. Reference samples prepared from lyophilized HepG2 cells were measured in parallel throughout the entire analytical process (technical replicates). Data from each metabolite in each sample were normalized against the median of the same metabolite in all reference samples on same plate to give normalized ratios. Lyophilized HepG2 cells reference samples were used to account for variability between plates and in concentration series for linearity checks. After 48 h, the assays were stopped by washing the wells once with 100 μL 0.9% NaCl followed by snap freezing the plates on liquid nitrogen for 5 s. Plates were placed immediately on dry ice and 50 μL ice-cooled isopropanol 80% (v/v) were added to quench metabolism and precipitate proteins. Plates were stored at − 80 °C until LC/ MS analysis.

### LC-MS/MS metabolomics

Metabolite profiling of cells was performed directly in the same 96-well plate according to a standardized protocol described below. In order to prevent any interaction with the cell material, the automatic sampler was adjusted to pick up the sample from the supernatant at a specified depth avoiding the contact with the bottom of the plate.

After the initial quenching step (50 μL isopropanol 80%), additional 70 μL of isopropanol 80%, containing internal standards (methionine-D3, tryptophan-D5, arginine-13C6-15N4, Boc-Ala-Gly-Gly-Gly-OH, coenzyme Q1, coenzyme Q2, coenzyme Q4) were added to each well. The internal standards were used for quality control by visual inspection (signal intensity, peak shape, retention time); they were not used for normalization. Afterwards, plates were shaken for 5 min, 750 rpm at 20 °C, and placed for 30 s in the ultrasonic device. Then, the plates were centrifugated for 10 min, at 5485 g, 15 °C. Further, 2.5 μL of the extract were injected each for reversed-phase and hydrophilic interaction liquid chromatography followed by MS/MS detection (AB Sciex QTrap 6500+) using the positive and negative ionization mode. For reverse-phase high-performance liquid chromatography (RP-HPLC, Ascentis Express C18, 5 cm × 2.1 mm, 2.7 μm Supelco), gradient elution was performed with mobile phase A, water/methanol/0.1 M ammonium formate (1:1:0.02, w/w), and B, methyl-tert-butylether/2-propanol/methanol/0.1 M ammonium formate/formic acid (4:2:1:0.07:0.035, w/w) 5.9 min linear gradient: 0 min 100% A, 0.5 min 75% A, 5.9 min 10% A; followed by 0% A until 6.7 min; re-equilibration at 100% A until 7.7 min; flow rate 600 μL/min; column temperature 40 °C HILIC (ZIC-HILIC, 2.1 × 100 mm, 3.5 μm, Supelco) gradient elution was performed with mobile phase C, acetonitrile/water (99:1, v/v) with 0.2% (v) acetic acid, and D, 7 mM ammonium acetate with 0.2% (v) acetic acid (5.0 min linear gradient: 0 min 100% C, 5 min 10% C; followed by a linear gradient back to 100%C until 6.5 min; re-equilibration at 100% C until 7.5 min; flow rate 600 μL/min; column temperature 40 °C.). Two LC-MS systems with identical configuration were used for the analysis. Normalization to lyophilized reference samples (see below) was used to compensate for variation from between analytical batches. The efficiency of this normalization procedure was checked by principal component analysis (PCA), confirming that Pool samples from different analytical batches clustered together.

During the quality control process, parameters such as coverage, linearity (R2), variability (RSD), and blank contributions were evaluated for each metabolite. An analyte would pass the quality control check if the following thresholds were met.

Coverage > 80%, blank contribution < 40%, slope > 0, and any of the following conditions (a, b, c, d) regarding the linearity (R2) and variability (RSD): (a) linearity > 0.8, variability < 0.3 or (b) linearity > 0.64, variability < 0.3 or (c) linearity (R2) > 0.8, variability (RSD) < 0.6 or (d) linearity (R2) > 0.64, variability (RSD) < 0.6. When a metabolite failed to pass the quality control check, data for this metabolite were excluded.

In our targeted approach, a pre-defined set of metabolites are identified by their analytical parameters: polarity (lipid vs polar), MRM transition (m/z ratios), and retention time. To confirm the identity of a metabolite, samples were spiked with the pure metabolite during method development whenever possible. When metabolites were not commercially available, fragmentation patterns and library matching were used to determine the most likely identity of those metabolites. The corresponding metabolites were then marked as “plausible.” Metabolites listed as “unknown” have well-defined analytical parameters but unknown chemical identity.

The corresponding chromatography techniques, ionization modes, Q1 mass [m/z], Q3 mass [m/z], ChEBI ID, ChEBI name, and MRM parameters for the measured metabolites is provided in Suppl. Fig. 1.

### Metabolomics data analysis

Reference samples derived from lyophilized untreated HepG2 cells (technical control samples) were measured in parallel throughout the entire analytical process. Data were normalized against the median of these lyophilized HepG2 cells reference samples, to give normalized ratios (performed for each sample per metabolite). This compensated for inter- and intra-instrumental variation. To correct for small differences in cell numbers within and between different treatment groups, data were also normalized to the within sample median, as described in detail by (Ramirez et al. 2018). For intracellular metabolomic analysis, the median of each sample was calculated across all the 221 measured metabolites.

To generate metabolic profiles for the different treatments, heteroscedastic *t* test (Welch test) was applied to log-transformed normalized metabolite data to compare treated groups with their respective controls.

To investigate the experimental variability, the variance of every log-transformed metabolite for both lyophilized HepG2 cells reference samples (technical replicates) and vehicle control samples was calculated (cells exposed to vehicle control during the assay time). These variances were back-transformed to linear scale, yielding a relative standard deviation (RSD) using the following formula:

$$\textrm{RSD}=1-{10}^{-\textrm{SDlog}}$$

Principal component analysis (PCA) and hierarchical clustering (HCA) analyses were performed using R software environment (https://www.r-project.org/). PCA was conducted using the ropls package (Thévenot et al. 2015) with log10-transformed input data and standard scaling. HCA was performed using the pvclust package (Suzuki et al. 2019), (https://CRAN.R-project.org/package=pvclust). Input: log10(Ratio), clustering method: Ward D2, distance method: Manhattan, bootstrapping: 10000 times.

## Results

### Method development and optimization

To develop a metabolomics in vitro assay compatible for high throughput, different parameters such as cell seeding density, influence of passage number, cytotoxicity testing, sample preparation, metabolite extraction, analytical method, and data processing known to have a significant impact on the cell metabolome were optimized in a 96-well plate format.

### Optimal cell seeding density determination

**Fig. 1:**
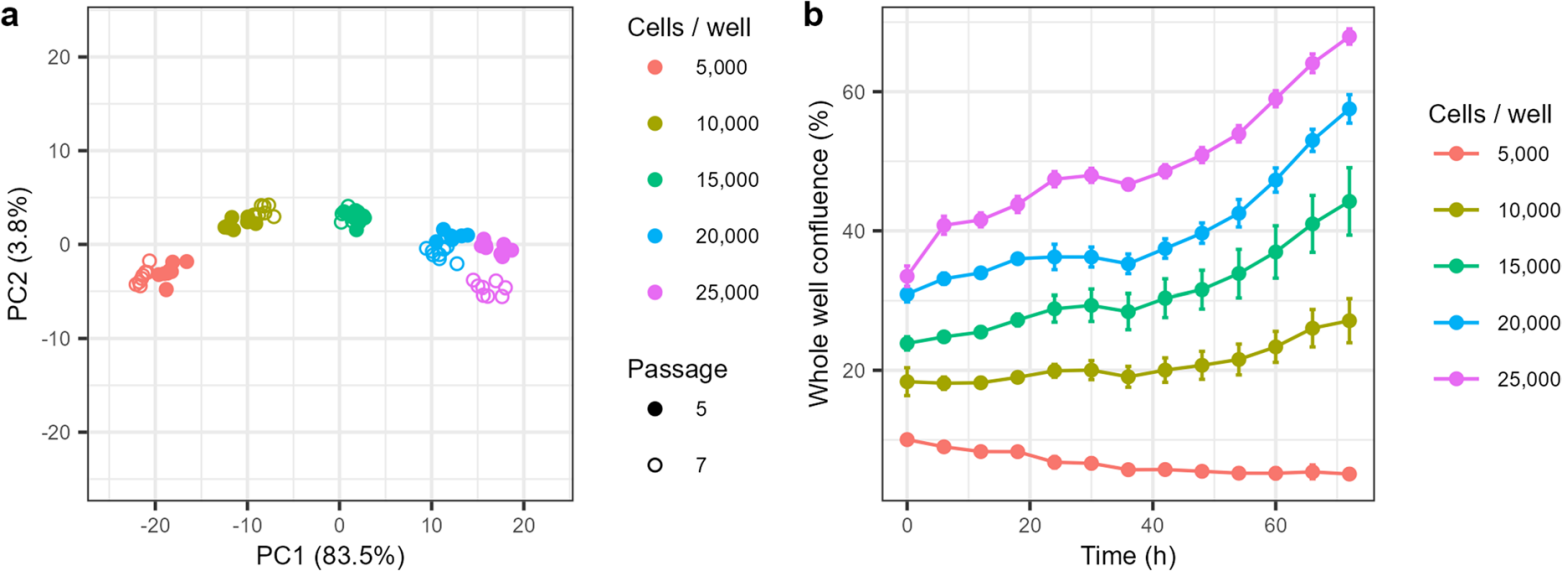
15,000 cells per well was selected as the optimal seeding number for the assay. **a** PCA analysis of the metabolic profiles of different cell seeding densities and passages (passage 5, passage 7). SAM normalization for cell number correction was not performed. **b** Cell confluence of different seeding densities during the time of the assay

To determine the optimal cell seeding density for the assay, different initial cell numbers ranging from 5000 to 25,000 cells/well were evaluated. Cells obtained from two different passage numbers (passage 5 and passage 7) were used for these experiments. Cell proliferation throughout the duration of the assay was monitored by measuring well confluence using real-time imaging analysis. After 48 h of incubation with vehicle control, the metabolic signal of vehicle control-treated cells was evaluated together with the well confluence (Fig. 1). Principal component analysis (PCA) of metabolic profiles showed that the initial cell seeding number was a strong driver for the separation along PC1, accounting for the 83.5% of the metabolic variation (see Fig. 1a). A separation of cells derived from passage 5 and passage 7 was evident in PC2, yet only accounting for a very minor fraction of the overall variability (3.8%). A comparably low peak intensity was observed at the initial cell seeding density of 5000 cells/well. Metabolic profiles of 20,000 and 25,000 cells/well were found close to each other in the PCA plot, suggesting a potential saturation for some metabolites around these cell numbers. Initial seeding densities of 10,000 cells/well and 15,000 cells/well exhibited a strong metabolic signal within the linearity range of the metabolic response and presented a relatively low within samples variability. However, seeding densities up to 10,000 cells/well showed decreased proliferation rates when compared with higher seeding densities (Fig. 1b). Further, 15,000 cells/well exhibited a strong metabolic signal, low variability, and a regular HepG2 cell proliferation rate and therefore was selected as the optimal initial cell seeding density to perform metabolomics experiments. PCAs and their corresponding loading plots before and after applying a cell number normalization procedure are shown in Suppl. Fig. 2. Considering the total duration of the assay (72 h) and the HepG2 doubling time (~ 30 h), the final cell numbers obtained with 15,000 cells/well was ~ 84,000 cells per sample.

### Influence of passage number on the metabolic response

**Fig. 2:**
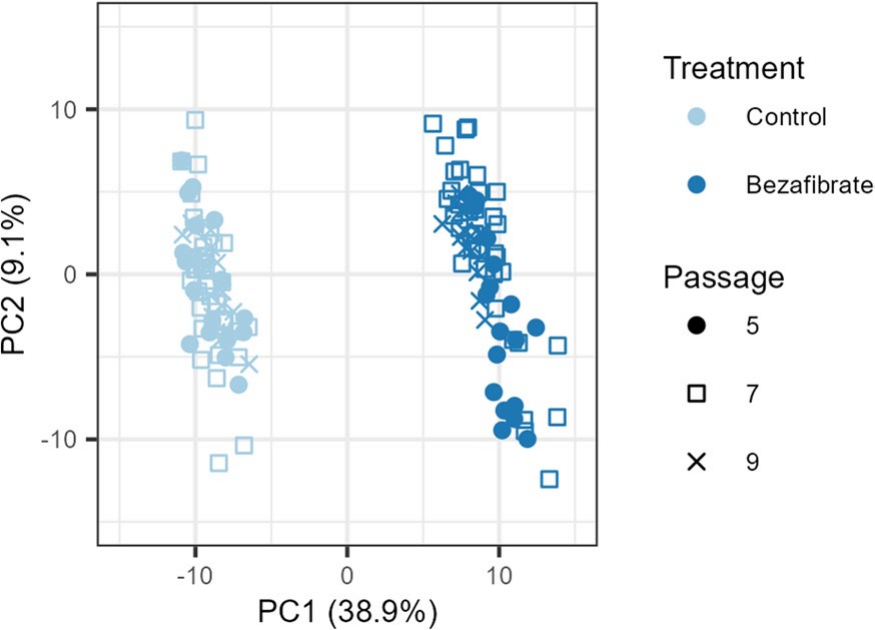
Bezafibrate-treated cells of different passages do not show a bias by experiment or cell passage. PCA analysis of the metabolic profiles of Bezafibrate-treated cells (positive control). Bezafibrate treatment showed a clear metabolic change compared to vehicle-treated cells. The results were obtained in 3 independent experiments using cell passages 5, 7, and 9

To evaluate the impact of passage number on the metabolome, cells derived from three different passages numbers (5, 7, and 9) were treated for 48 h with Bezafibrate, a substance that has served as a quality/positive control in metabolomics studies due to its pronounced and reproducible effect on the metabolome. Metabolic profiles of Bezafibrate-treated cells originating from different passage numbers were compared by PCA analysis (Fig. 2). Bezafibrate-treated samples were clearly separated from control samples. It was observed that the strongest effect was due to substance treatment, accounting for 39% of the variation in PC1. Results showed a high overlap among samples from different passage groups, indicating that cellular passage was not a major source of variation. Based on these data, different cell passages (from 5 to 9) could be used in the metabolomics in vitro assay, increasing the flexibility of the method without a major impact on the results.

### Proof of concept with test substances

Once the main parameters of the assay were established, its performance as liver toxicity MoA screening test was evaluated. Seven substances known to cause liver toxicity through three different MoAs were tested (peroxisome proliferation: Acifluorfen, Wy-14643, and Bezafibrate; liver enzyme induction: Pendimethalin, Aroclor, and β-naphthoflavone; liver enzyme inhibition: Ketoconazole).

### Cytotoxicity testing for dose selection

To select compound concentrations for the metabolome experiments, range finder experiments for each substance were carried out. After administering increasing concentrations of test compounds, cytotoxicity and cell viability were assessed (Suppl. Fig. 3). CellToxGreen assay measures cell death and therefore it was used to identify concentrations that were highly cytotoxic. ATP production, a more sensitive endpoint and likely to be a closer proxy to impairments in cellular metabolism, was used to generate dose response curves for each substance and estimate effective concentration (EC1, EC5, EC15, EC50, EC85) values. Based on ATP-estimated EC values (Table 2), five concentrations (C1–5) covering the full dose response range were selected per substance for the metabolome experiments (see Suppl. Fig. 4). To experimentally assess cytotoxic effects of selected doses, cell viability and cytotoxicity were measured in parallel with metabolomics experiments in plates handled and treated exactly as the ones used for metabolomics (Table 3, Suppl. Fig. 5). Cells treated with C1 and C2 exhibited a percentage of viability of ≥ 97% for all substances, except for Aroclor 1254 where the viability for C1 was 95.5% and for C2 was 80.5%. C3 had a stronger effect on the viability (≥ 80%) for all substances except for Aroclor 1254 (60.6%) and Bezafibrate (71–84%). As expected, C4 and C5 of all substances had a pronounced effect on the cellular viability ranging from 34 to 65% for C4 and from 3 to 28% for C5.

**Table 3:** Cell viability in metabolomics experiments. Percentage of viability (ATP) compared to the vehicle control (*n* = 6). *Bezafibrate 1000 μM was used as a positive control in each experiment. Actual concentrations corresponding to the C1–C5 of each substance can be found in Table 2 of materials and methods

Substance	C1 (%)	C2 (%)	C3 (%)	C4 (%)	C5 (%)	Bezafibrate* (%)
Acifluorfen	104.7 ± 4.2	105.2 ± 4.8	99.9 ± 1.3	58.4 ± 1.5	27.8± 1.3	84.1 ± 2.3
Wy-14643	100.9 ± 3.8	97.1 ± 3.1	94.6 ±1.8	46.4 ± 2.0	23.2 ± 1.1	76.3 ± 3.2
β-naphthoflavone	103.4 ± 4.3	113.8 ± 3.7	104.1 ± 1.6	34.5 ± 3.4	15.0 ± 2.1	75.0 ± 1.5
Aroclor 1254	95.5 ± 4.1	80.5 ± 5.7	60.6 ± 7.5	8.7 ± 1.6	3.9 ± 3.2	71.8 ± 2.8
Pendimethalin	98.3 ± 3.9	100.3 ± 2.9	82.4 ± 4.9	36.1 ± 3.2	18.5 ± 3.2	78.0 ± 0.7
Ketoconazole	102.4 ± 3.1	102.0 ± 0.9	91.2 ± 1.5	64.7 ± 1.5	28.2 ± 1.9	79.3 ± 2.2

### Metabolomics

After treatment, intracellular metabolites were extracted for semiquantitative targeted metabolomics via LC-MS/MS. 221 unique analytes were measured of which 156 were annotated and 65 remained unknown. Annotation was done during method development, using different approaches such as spiking with reference compounds or LC-Q-Tof-HRMS analysis for matching the results with library data or with results from similar compounds of the same compound class. Measured metabolites were grouped into 12 ontology classes. An enrichment analysis was carried out to evaluate the number of significantly changed metabolites per ontology class (Suppl. Fig. 6). The data revealed dose dependency in all cases, with increasing number of altered metabolites at higher concentrations.

### Experimental variability and reproducibility

The experimental variability of the technical (lyophilized reference samples) and biological controls (vehicle treated cells) demonstrated the robustness and reproducibility of the method. Control samples revealed RSD values of 8% to 11%. The technical replicates had an RSD of 8% to 10% (Suppl. Fig. 7). Reproducibility was also evaluated under treatment conditions using the results of Bezafibrate, which was included as positive control in all the plates. The metabolome profile of different Bezafibrate experiments clustered together in the PCA of all analyses, indicating the homogeneity of the samples and experiments. Moreover, the univariate analysis of the Bezafibrate treatment effects demonstrated consistent pattern of metabolite changes across all plates.

### PCA analysis reveals dose response effect by metabolomics

**Fig. 3:**
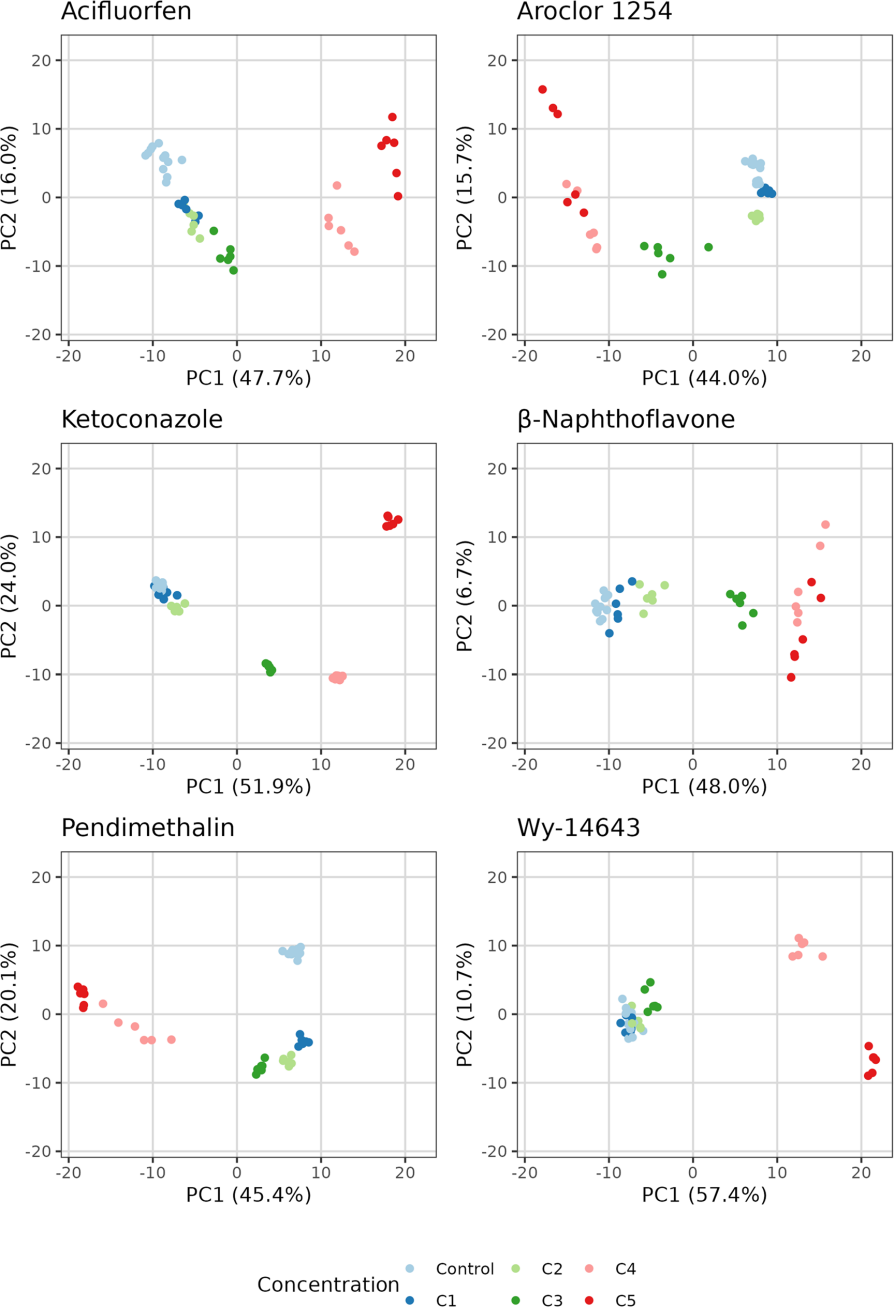
PCAs of metabolomics dose-response effect. PCA analysis of the metabolic profiles of **a** Acifluorfen, **b** Wy-14643, **c** β-naphthoflavone, **d** Aroclor, **e** Pendimethalin, and **f** Ketoconazole-treated cells at five different concentrations. Actual concentrations corresponding to the C1–C5 of each substance can be found in Table 2 of materials and methods. Bezafibrate was used as a positive control in all plates (data not shown)

Metabolite profiles were further analyzed by PCA. Concentration-dependent responses were observed for each substance (Fig. 3). For pendimethalin, aroclor, acifluorfen, and ketoconazole, low (C1 and C2) and intermediate concentrations (C3) were separated from control samples with a similar dose response trajectory: the initial separation from controls at the lowest concentrations is visible in a PC2 response followed by increasing PC1 separation at higher concentrations. For ß-Naphthoflavone, all concentrations were quantitatively different but separated from control samples in the same direction (PC1). For Wy-14643, C1 and C2 clustered together with controls, the intermediate level (C3) separated along the PC2, and high concentrations clearly separated along the PC1. In summary, low and intermediate concentrations drift away from controls in the same direction in a dose response manner while high concentrations exert a strong effect on the metabolome (PC1) and separate in a different direction suggesting a different impact of middle and high concentrations levels on the metabolome. These results show that (1) a metabolome-based dose response can be obtained and (2) metabolite profiles resulting from cytotoxic effects on the metabolome are distinguishable from specific substance-related effects; (3) metabolomics is more sensitive than ATP measurement; and significant metabolite changes were already observed at concentrations that caused no reductions in ATP levels (e.g., C1, C2).

### Differentiation of hepatotoxicity modes of action

**Fig. 4:**
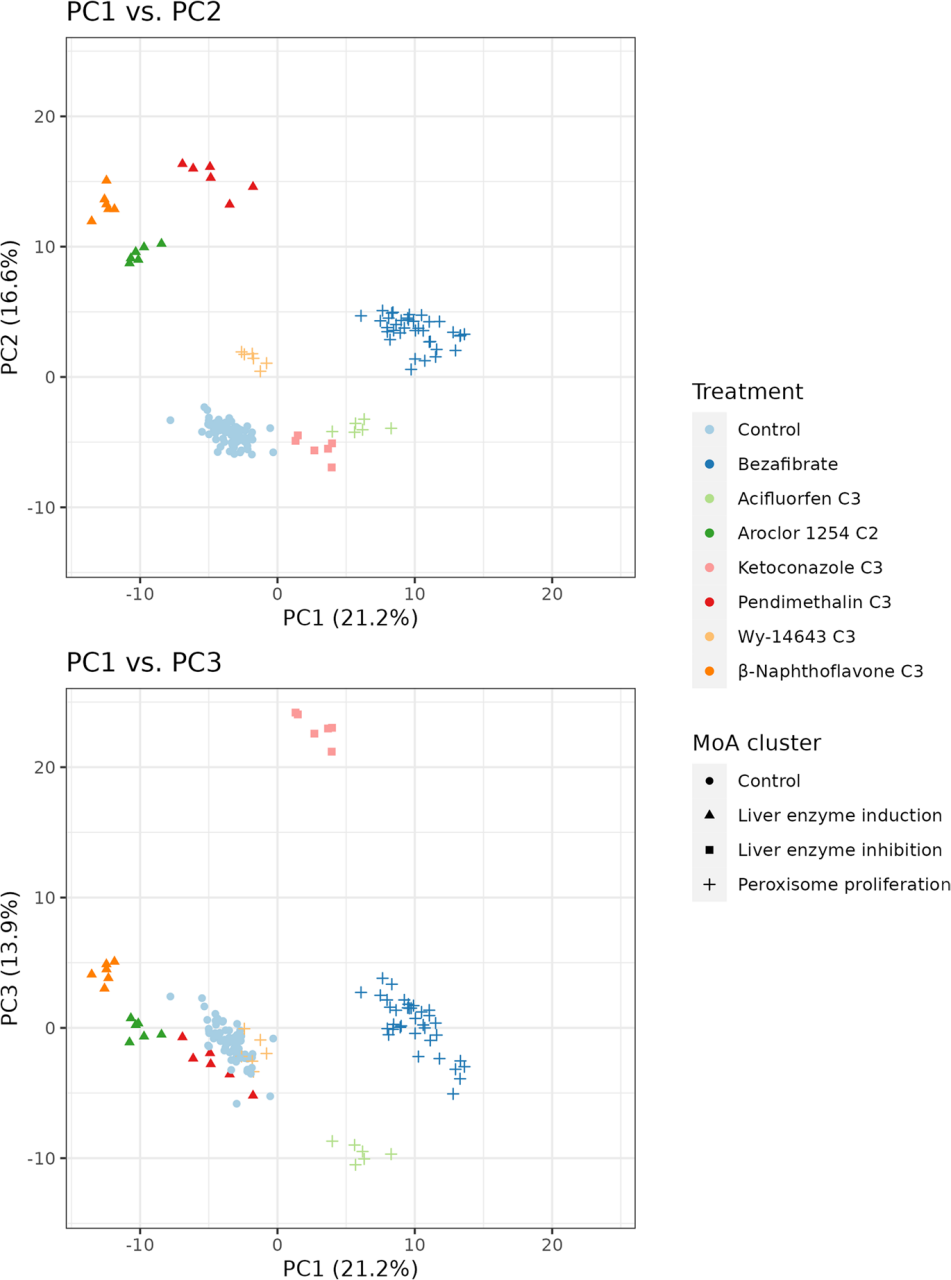
PCA of metabolic profiles shows a MoA-specific clustering of liver toxicants. PCA of metabolite profiles of HepG2 cells treated for 48 h with three liver enzyme inducers (pendimethalin, aroclor, β-naphthoflavone), three peroxisome proliferators (bezafibrate, acifluorfen, wy-14643) and one liver enzyme inhibitor (ketoconazole) allows to discriminate between the different mode of actions of these substances. Intermediate concentrations (C3 for pendimethalin, β-naphthoflavone, acifluorfen, wy-14643, and ketoconazole and C2 for aroclor) were selected for the analysis. Upper panel PC1 vs. PC2; lower panel PC1 vs. PC3

A meaningful metabolomics-based assessment of MoA requires a concentration high enough to cause biochemical alterations but not excessive to induce extensive cell damage and lethality. Intermediate concentration levels (C3 for pendimethalin, β-naphthoflavone, acifluorfen, wy-14643, and ketoconazole and C2 for aroclor) were clearly distinguishable from controls in the PCA (Fig. 3), which showed a mild reduction of cell viability (less than 10%) and presented low cytotoxicity (Suppl. Fig. 5); therefore, were selected for further analysis. Applying a PCA, a separation by mode of action was observed (Fig. 4). Treated samples were separated from vehicle controls in PC1, accounting for 21% of the total variation. Three different clusters corresponding to the evaluated MoAs were detected. The separation of the peroxisome proliferators cluster (acifluorfen, bezafibrate and wy-14643) and liver enzyme inducers cluster (aroclor, β-naphthoflavone, and pendimethalin) was visible in PC2, representing 17% of total variation. The separation of liver enzyme inhibitor (ketoconazole) was distinguishable in PC3 which accounted for 14% of the total variation. A 3D PCA plot can be found in the supplemental information (Suppl. Fig. 8).

**Fig. 5:**
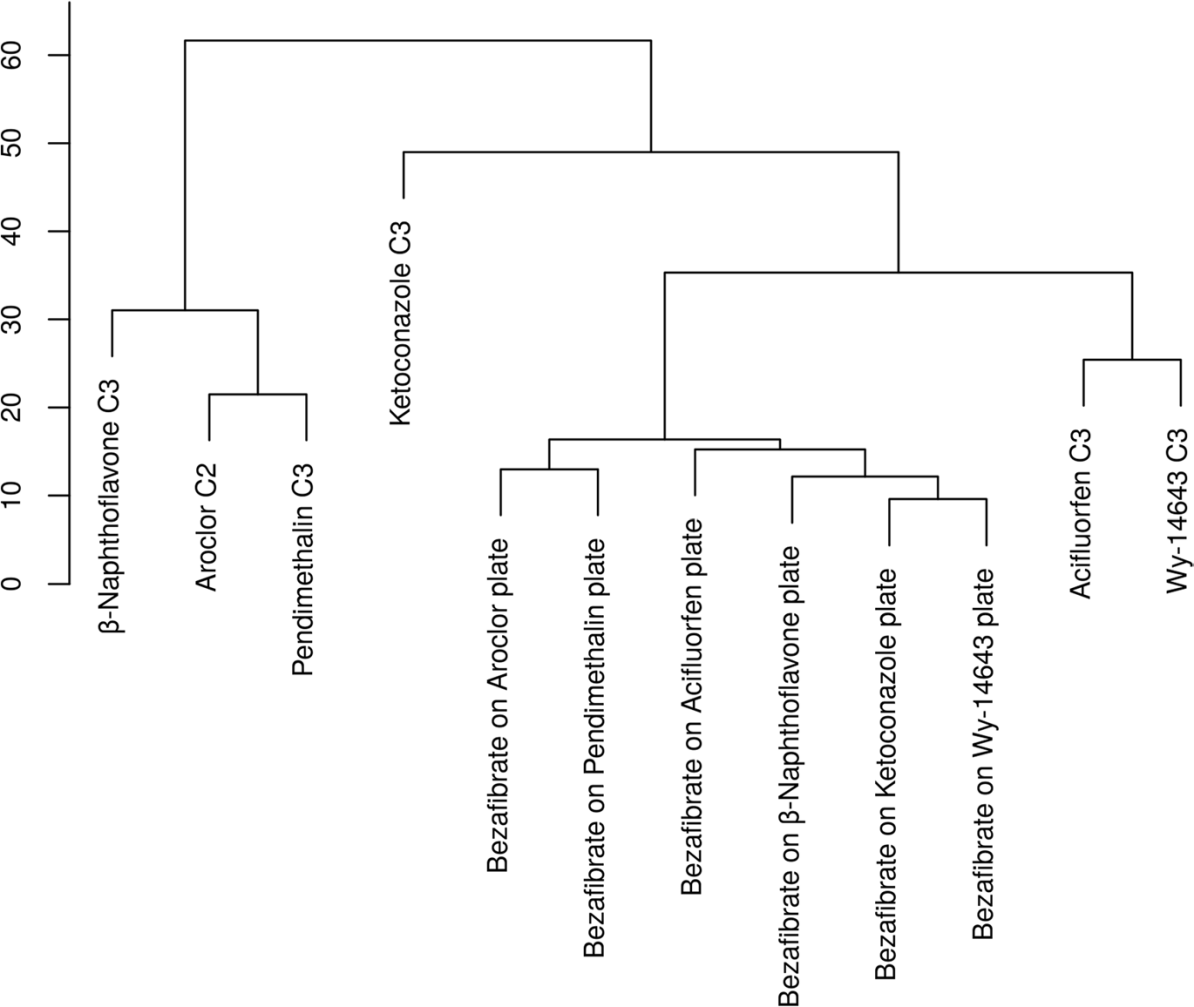
Hierarchical clustering analysis of metabolic profiles shows a MoA-specific clustering of liver toxicants. HCA of metabolite profiles of HepG2 cells treated for 48 h with three liver enzyme inducers (Pendimethalin, Aroclor, β-Naphthoflavone), three peroxisome proliferators (Bezafibrate, Acifluorfen, Wy-14643), and one liver enzyme inhibitor (Ketoconazole) allows to discriminate between the different mode of actions of these substances. Intermediate concentrations (C3 for Pendimethalin, B-naphthoflavone, acifluorfen, Wy-14643, and Ketoconazole and C2 for Aroclor) were selected for the analysis. Input: log10(Ratio), clustering method: Ward D2, distance method: Manhattan, bootstrapping: 10,000 times

A hierarchical clustering analysis (HCA) further confirmed the compounds clustering by MoA (Fig. 5). These results indicate that 96-well plate in vitro metabolic profiling can distinguish between different liver toxicity MoAs.

### Characteristic metabolite changes for each MoA

The metabolites set of significantly altered metabolites can give a better mechanistic understanding of liver toxicity MoAs. Therefore, intracellular metabolomes of cells treated with the intermediate concentration (C3 for pendimethalin, β-naphthoflavone, acifluorfen, wy-14643, ketoconazole, and C2 for aroclor) were then evaluated at a single metabolite level and used to identify unique sets of altered metabolites for each MoA. Metabolite sets that were potentially relevant for a specific MoA were generated by comparing metabolite profiles of substances belonging to the same MoA and selecting all commonly changed significantly increased or decreased metabolites.

Peroxisome proliferators (bezafibrate, acifluorfen, wy-14643) showed a decrease in concentrations of short (propionylcarnitine) and medium (hexanoylcarnitine, acylcarnitines) carnitines, ketoleucine, taurine, creatine, 5-hydroxytryptophan, s-adenosylhomocysteine, deoxycytidine, glycerol-3-phosphate, and higher concentrations of *N*-acetlyaspartate and some triacylglycerols. Additionally, concentrations of phospholipids, ceramides, and sphingomyelins were decreased after treatment (Suppl. Fig. 9).

Cells treated with the enzyme inducers aroclor, pendimethalin, and β-naphthoflavone showed increases of tyrosine, the redox carriers flavin adenine dinucleotide (NAD) and glutathione (GSH) and the glutathione precursor cysteinylglycine. In addition, enzyme inducers were characterized by reductions of proline, myo-inositol, *N*-acetylglucosamine, deoxycytidine, the carnitine derivative *O*-acetyl carnitine, short and medium chain carnitine derivatives, and several triacylglycerols (Suppl. Fig. 10).

The metabolite profile of ketoconazole, representative of the liver enzyme inhibitor MoA, exhibited lower concentrations of amino acids and related metabolites, the redox carriers GSH, pyroxidal and coenzyme Q10, ceramides and cholesteryl esters, and increases in levels of long chain carnitine derivatives, taurine, choline, and sphingomyelin (Suppl. Fig. 11).

In summary, our results show that different MoA were characterized by specific metabolites as common denominators for the respective MoA. Additionally, reduced levels of creatine, carnitine, and pantothenic acid and increased levels of lysophosphatidylcholine and lysophosphatidylethanolamine were found to be metabolite alterations shared by the 3 MoAs; consequently, these metabolites cannot be regarded as MoA specific but rather general for hepatotoxicity.

## Discussion

### Method development and optimization

Several potentially influencing factors for the development of a high-throughput in vitro metabolomics technique have been evaluated and optimized.

### Cell seeding density determination

Miniaturizing an essay to a 96-well format implicates reductions in cell numbers thus limiting the available biomass for analysis. Therefore, an important issue was to select an appropriate initial cell density. Based on the assessment of cell growth and metabolomics signal strength of different cell seeding densities, 15,000 cell/well provided best results.

Our results demonstrate that seeding density has a major effect on the cellular growth rate and consequently on cell numbers at the end of the experiment. It has been shown that dose response metrics such as IC50 are influenced by growth rates (Hafner et al. 2016). Therefore, optimizing seeding densities and reporting final cell numbers is critical in assay development to reduce interexperimental variability and improve the replicability and reproducibility of dose response curves (Larsson et al. 2020).

### Influence of passage number on the metabolic response

Flexibility is a crucial parameter for implementing an in vitro assay. In industrial settings, studies are designed for serial running of large numbers of samples in wide ranges of experimental conditions, making the use of a single, cell passage impractical. Yet, using different passage numbers can represent a source of variability that could reduce the statistical significance of the analysis (Moreno-Torres et al. 2021). We evaluated passage number (passage 5–9) as a possible confounding factor and showed that it has no significant impact on the metabolome, increasing the flexibility of the assay without impacting the biological interpretation. A similar observation was made by (Moreno-Torres et al. 2021) who showed that cell passage has a minor contribution (6%) to the data variance in the PCA.

### Sample extraction

To avoid complex sample manipulation, we have implemented a one-phase liquid extraction using isopropanol. Monophasic extractions using alcohols (mostly methanol) are usually sufficient to remove most macromolecules such as proteins and nucleic acids, avoid selection bias, cover a wide range of polar to non-polar metabolites and in contrast to other organic solvents, are compatible with polystyrene (96 well-plate material) (Andresen et al. 2022). In comparison with methanol, isopropanol allows better protein precipitation, has a higher boiling point, which reduces sample evaporation and offers a broad polarity range (Andresen et al. 2022). Metabolite extraction was done directly in 96 well-plates, avoiding cell scrapping, trypsinization or additional steps which cause cellular-perturbations or biomass loss (Bordag et al. 2016; Dubuis et al. 2018; Zampieri et al. 2018).

### Optimization of the metabolome coverage by using LC-MS

In this study, we focused on using LC-MS as a single analytical technique and combined HILIC (for polar metabolites) and RP (optimized for lipid species) chromatography to expand the metabolome coverage. The results revealed that a wide range of metabolites from highly polar (amino acids, nucleobases, cofactors) to lipidic (lysoPLs, PLs, TGs) was covered. Lipid metabolites accounted for 75% of the 221 annotated features. Therefore, even though we increased the LC spectrum significantly, the detection of polar metabolites was limited. Our current method could potentially be improved by optimizing the HILIC protocol as shown by (Gerdemann et al. 2022), by adding pre-column derivatization steps (Walvekar et al. 2018) or by implementing an additional method for energy metabolism metabolites (Balcke et al. 2011). However, these additional sample preparations and the need of different aliquots would increase the experimental time and cost. Therefore, from a practical point of view, our experiments showed that performing the analysis using a single sample preparation and analytical condition represents a good tradeoff between simplifying the system and still getting sufficient information to discriminate between different MoAs.

### Proof of concept: MoA differentiation

HepG2 cells have been instrumental for investigating the molecular and cellular processes involved in hepatotoxicity. Although their limited drug metabolizing and transport capabilities are well acknowledged, their low cost, high reproducibility, and human origin make them a suitable option for initial screenings and compound prioritization.

The applicability of our system to differentiate between MoAs was tested with seven substances known to be representative of three different liver toxicity MoAs (Peroxisome proliferation, liver enzyme induction, and liver enzyme inhibition). Five doses covering a wide range of concentrations from EC1 to EC85 (intracellular ATP) were used. A clear metabolomics-based dose response was observed by both multivariate and univariate analysis (Fig. 3, Suppl. Fig. 6).

Noteworthy, the dose selection was based on estimated EC values obtained from ATP-based dose response curves in the range finder experiments. The estimated EC values did not correlate exactly with the experimental ATP measured values (Table 3). This was highly dependent on the compound and might be related to the specific dose-response nature of each substance. Expanding the number of tested concentrations in the range finder experiments would enable to build improved dose-response curves that allow for more accurate EC estimations. Yet, ATP measurement represents a highly sensitive and metabolic-related endpoint and therefore represents a suitable approach for metabolomics dose setting.

Then to evaluate the specific MoA-related metabolomics response and exclude confounding cytotoxic effects, we selected a concentration that (1) did not cause any significant cell death (assessed by CellToxGreen assay) and (2) induced a slight to moderate loss of ATP (ATP level decreased by less than 20% as compared to controls). In most cases, this turned out to be the C3 concentration. The results of the PCA analysis at this concentration demonstrated a good separation between peroxisome proliferators, enzyme inducers, and the enzyme-inhibiting compound.

Early identification of MoA is a powerful biologically driven classification tool essential in compound development. The next step would be to build a large database which would broaden the spectrum of covered MoAs, similar to our in vivo metabolomics database (MetaMap®Tox) (Van Ravenzwaay et al. 2015). Thus, following the metabolome analysis of a new compound, a PCA comparison with that of a reference compound (i.e., compounds with a known MoA) may help to quickly identify the probable MoA and offer the possibility of biological-based read across analysis.

### Non-specific markers of hepatotoxicity

Following the PCA comparison, metabolic profiles of subtoxic doses were subjected to univariate statistics to identify individual metabolite changes. Concentrations of carnitine and pantothenic acid were significantly decreased and levels of lysophosphatidylcholines (LPC) and lysophosphatidylethanolamines (LPE) significantly increased in all treatments. These metabolic changes were observed irrespective of the substance MoA and therefore can be considered as nonspecific, general markers of hepatoxicity.

Pantothenic acid is an essential nutrient required to synthesize coenzyme A (CoA) (Leonardi et al. 2007). During fatty acid degradation, CoA and carnitine are required to activate and mobilize long chain fatty acids into the mitochondria via the carnitine shuttle. Lower intracellular concentrations of carnitine and pantothenic acid suggest a higher mobilization of lipids into the mitochondria for β-oxidation. Reduced concentrations of these two metabolites have been reported consistently in in vitro studies after exposure to hepatotoxicants such as 2,3,7,8-tetrachlorodibenzo-*p*-dioxin (TCDD) (Ruiz-Aracama et al. 2011), peroxisome proliferators (Ramirez et al. 2018), sodium valproate (Cuykx et al, 2018a), and dichloroacetate (Dubuis et al. 2018). These metabolite changes point toward a general imbalance of the cellular energy status and could be indicative of mitochondrion malfunction, a pathway which is frequently target of hepatotoxic compounds (Mihajlovic et al. 2022).

Elevated concentrations of both LPC and LPE represent an increased turnover of phospholipids species and are as well a common finding in investigations of liver pathologies and in the nephrotoxic-related MoA—mitochondrial DNA interaction—in kidney cells (García-Canaveras et al. 2011; Beyoglu et al. 2013; Birk et al. 2021).

### MoA-specific metabolite profiles

For each of the MoAs, panels of specific combinations of intracellular metabolite changes were identified which are unique for the adverse outcome pathway.

Peroxisome proliferators (PP) are pharmaceutical and chemicals that increase the number and size of peroxisomes in vivo via the activation of the nuclear receptor PPARα which acts as a central regulator of hepatic lipid metabolism (Aoyama et al. 1998).

The metabolic profile of PP exhibited lower levels of short and medium acylcarnitines, ketoleucine, taurine, and creatine and higher concentrations of *N*-acetylaspartate and some triacylglycerols. Long-chain acylcarnitines are oxidized in the mitochondria and peroxisomes via β-oxidation to short- and medium-chain carnitines that can be subsequently utilized together with ketogenic amino acids for the formation of ketone bodies via the branched-chain amino acid (BCCA) metabolism. Lower levels of propionylcarnitine and hexanoylcarnitine together with decreased levels of the ketogenic amino acid ketoleucine support the increased synthesis of ketone bodies characteristic of an energy metabolism switch from glycolysis to fatty acid β-oxidation. Taurine plays an important role in lipoprotein metabolism (Yanagita et al. 2008) and has been shown to attenuate the effects of drug induced hepatic injury by acting as an antioxidant during lipid peroxidation (Mas et al. 2004; Murakami et al. 2018). A higher utilization of taurine due to increased β-oxidation could have resulted in the lower levels of the amino acid observed in PP-treated cells.

Lower levels of creatine and *S*-adenosylhomocysteine were also observed after PP treatment. It has been shown that some PP competes for the same binding site on PPAR-α as homocysteine (Hunt et al. 2002). In addition, creatine administration is known to decrease the homocysteine production in liver, preventing fat accumulation and resulting in beneficial effects in fatty liver and non-alcoholic liver disease (Barcelos et al. 2016).

Increased levels of the acetyl-CoA precursor *N*-acetyl-aspartate (NAA) could have resulted as a consequence of the excess of acetyl-CoA produced by high rates of fatty acid oxidation in PP-treated cells (Prokesch et al. 2016).

Metabolic profiles of enzyme inducers showed decreased levels of proline. Proline can be used by cancer cells as energy source and/or as precursor of protein synthesis. Recent studies have demonstrated that proline participates in the regulation of redox balance and energy status (Zheng et al. 2021). Concentrations of the glucose derivates myo-inositol and *N*-acetylglucosamine were as well reduced after treatment. Myo-inositol is implicated in the modulation of glucose metabolism through its role in insulin signal transduction (Bevilacqua et al. 2018). These changes together with lower concentrations of the carnitine precursor o-acetylcarnitine, short and medium carnitine derivates, and several triacylglycerols suggest an alteration of the cellular energy balance.

Increased concentrations of the glutathione precursor cysteinylglycine as well as the redox carriers flavin adenine dinucleotide (FAD) and glutathione (GSH) indicate the activation of the antioxidant response. CYPs enzymes induction plays an important role in increased hepatic clearance but also contributes to the formation of chemically reactive metabolites that can lead to toxicity. However, enzyme induction by itself is generally viewed as a compensatory and not an adverse response (Mattes et al. 2014). In line with our observations, the elevation of glutathione and its precursor cysteinylglycine suggest a stimulation of de novo synthesis of glutathione indicative of an early cellular response to counteract the ROS production generated by higher activities of CYPS enzymes.

Ketoconazole is an imidazole fungicide that acts as potent inhibitor of the human CYP 3A4 enzyme and was used in this study to assess the metabolic profile of liver enzyme inhibition. Cells exposed to Ketoconazole showed lower concentrations of amino acids (threonine, proline, and glutamate), amino acid-related metabolites, antioxidants (coenzyme Q10, pyroxidal, GSH), and ceramides, together with an increase in the levels of long-chain acylcarnitines (tetradecanoylcarnitine, hexadecenoylcarnitine, hexadecanoylcarnitine, octadecenoylcarnitine), taurine, and sphingomyelins.

The accumulation of taurine and long-chain acylcarnitines indicates an inhibition of mitochondrial fatty acid β-oxidation accompanied by an increased protein catabolism as an alternative for energy production. Reduced levels of antioxidant molecules such as GSH and its precursor glutamate indicate oxidative stress. In line with our findings, HepG2 and HepaRG cells exposed to Ketoconazole and other antimycotic azoles showed a reduction in the mitochondrial membrane potential and impaired activity of the electron transport chain. As a consequence, increased production of reactive oxygen species (ROS) was generated leading to mitochondrial oxidative stress (Haegler et al. 2017). Similar metabolic alterations were reported after exposing HepG2 cells to the hepatotoxic carcinogen TCDD. TCDD and dioxin-like chemicals have been shown to inhibit human CYP1A2 activity in vitro which could explain the similarity in their metabolic profiles (Staskal et al. 2005).

The observed reduction in ceramides and increased concentrations of sphingomyelins suggest a higher turnover of ceramides. The sphingolipid metabolism is closely linked to inflammation and the downregulation of ceramides is associated with the development and progression of different liver pathologies (Tanase et al. 2021).

As we have used only one compound representative for the MoA of CYP 3A4 enzyme inhibition, additional compounds need to be tested to verify the specific nature of the metabolome profile.

Taken together, our results are in agreement with previously described biochemical changes of the compounds tested and, although not all intracellular metabolites were measured, the combination of altered metabolites was found to be sufficient to differentiate between hepatotoxicity MoAs and shed light into the mechanisms of toxicity.

Importantly, one of the key aspects of toxicity of twenty-first century relies in dose response modeling (Andersen et al. 2009). However, current screens are usually done only in few concentrations, hampering the calculation of meaningful dose response metrics such as IC_50_ and consequently limiting the applicability of in vitro systems in risk assessment (Olesti et al. 2021). By escalating the throughput, our system allowed to cover key points of the dose response curve from very mild effects (EC_1_) to overt toxicity (IC_85_). The potential of omics technologies for determining point of departure (PoD) has been highlighted (Thomas et al. 2007; Kang et al. 2020). Recently, a concentration-response analysis derived from in vitro metabolomics was used for benchmark concentration (BMC) modeling, showing to be a sensitive and quantitative indicator of liver injury potential (Crizer et al. 2021). High-throughput metabolomics methods that allow for multiple dose testing can contribute to fast screening that directs additional studies based on PoD estimations.

Finally, strategies to induce and manipulate the gene expression of multiple CYPs enzymes in HT systems have been described (Kwon et al. 2014) and could be applied to our HepG2 platform to expand its applicability. Additionally, improved standardization and cost reduction of metabolically more competent cells (e.g., HepaRG or hiPSCs) would allow to expand their implementation in HT systems (Mirahmad et al. 2022).

## Conclusion

We have designed a highly reproducible 96-well-plate targeted in vitro metabolomics platform and optimized critical experimental parameters for rapid and cost-efficient hepatotoxicity screening. The system was tested with seven model compounds representative of three different liver toxicity MoAs demonstrating the applicability of the assay to reproduce metabolomics dose response effects, distinguish between different liver toxicity MoAs, and identify key metabolites and patterns indicative of general and MoA specific hepatotoxicity.

Identifying the MoA of a compound in the early stage of compound development can guide the selection of the most prominent leads, help to identify unwanted effects, and provide a valuable foundation for a more targeted hazard assessment. Due to resources reduction and throughput increase, this assay allows to assess a broader range of concentrations that would enable a more accurate metabolome-based PoD, in vitro to in vivo extrapolations (IVIVE), and substance kinetic analysis.

This method can be extended to further cell lines and iPSCs for the investigation of different organ toxicities and is suitable for a wide range of screening applications that demand rapid, cost effective, and high throughput analysis.

### Supplementary information

ESM 1 (PDF 1828 kb)

## Data Availability

The datasets generated during and/or analyzed during the current study are available from the corresponding author on reasonable request.
