# Supplementary material for: A high-throughput metabolomics in vitro platform for the characterization of hepatotoxicity
Source: Cell Biol Toxicol. 2023 May 4;39(6):2899–917. doi: 10.1007/s10565-023-09809-6 (PMC10693528; doi:10.1007/s10565-023-09809-6)
Supplement: Supplementary file 1 — (PDF 1828 kb) [file 10565_2023_9809_MOESM1_ESM.pdf]

A High Throughput Metabolomics In *vitro* Platform for The Characterization of Hepatotoxicity

Cell Biology and Toxicology

Sabina Ramirez-Hincapie[1], Barbara Birk[1], Philipp Ternes[2,] Varun Giri[1], Volker Haake[2], Michael Herold[2], Franziska Maria Zickgraf[1] Andreas Verlohner[1], Hans-Albrecht Huener[1], Hennicke Kamp[2], Peter Driemert[2], Robert Landsiedel[1,3], Elke Richling[3], Dorothee Funk-Weyer[1], Bennard van Ravenzwaay[5]

[1] BASF SE, Experimental Toxicology and Ecology, Ludwigshafen, Germany

[2] BASF Metabolome Solutions GmbH, Berlin, Germany

[3] Free University of Berlin, Pharmacy, Pharmacology and Toxicology, Berlin, Germany.

[4] Food Chemistry and Toxicology, Department of Chemistry, University of Kaiserslautern - Landau, Kaiserslautern, Germany

[5] Environmental Sciences Consulting, Altrip, Germany

E-mail corresponding author: sabina.ramirez-hincapie@basf.com

Supplementary information

Suppl Fig. 1. Mass spectrometry parameters. *corresponding chromatography techniques, ionization modes, Q1 mass [m/z], Q3 Mass [m/z],ChEBI ID, ChEBI name, and MRM parameters for the measured metabolites*

Metabolite Name	Metabolite Class	Chromatography	Ionization	Q1 Mass [m/z]	Q3 Mass [m/z]	ChEBI ID	ChEBI Name
Glutamate	Amino acids	HILIC	ESI negative	145.9	101.7	CHEBI:18237	glutamic acid
Proline	Amino acids	HILIC	ESI negative	114	67.9	CHEBI:17203	L-proline
Taurine	Amino acids	HILIC	ESI negative	124	80	CHEBI:15891	taurine
Threonine	Amino acids	HILIC	ESI negative	117.9	73.9	CHEBI:16857	L-threonine
Tyrosine	Amino acids	HILIC	ESI negative	180	118.9	CHEBI:17895	L-tyrosine
5-Hydroxytryptophan	Amino acids related	HILIC	ESI positive	220.9	91	CHEBI:28171	5-hydroxytryptophan
Creatine	Amino acids related	HILIC	ESI negative	129.9	88.1	CHEBI:16919	creatine
Cysteinylglycine	Amino acids related	HILIC	ESI positive	178.8	76.1	CHEBI:4047	L-cysteinylglycine
Glycylleucine	Amino acids related	HILIC	ESI positive	189.1	86.1	CHEBI:73514	Gly-Leu
Ketoleucine	Amino acids related	RP-HPLC	ESI negative	129.2	129.2	CHEBI:48430	4-methyl-2-oxopentanoic acid
N-Acetylaspartate	Amino acids related	HILIC	ESI positive	175.9	134.1	CHEBI:21547	N-acetyl-L-aspartic acid
N-Acetylleucine	Amino acids related	HILIC	ESI positive	174.2	86.1	CHEBI:17786	N-acetyl-L-leucine
N-Acetylserine	Amino acids related	HILIC	ESI positive	148.1	106.2	CHEBI:45441	N-acetyl-L-serine
Pipecolic acid	Amino acids related	HILIC	ESI positive	129.9	84.1	CHEBI:17964	pipecolic acid
S-Adenosylhomocysteine	Amino acids related	HILIC	ESI positive	385.1	136	CHEBI:16680	S-adenosyl-L-homocysteine
myo-Inositol-2-phosphate	Carbohydrates and related	RP-HPLC	ESI negative	259.2	79.2	CHEBI:62383	1D-myo-inositol 2-phosphate
N-Acetylglucosamine	Carbohydrates and related	HILIC	ESI positive	222	203.8	CHEBI:506227	N-acetyl-D-glucosamine
Adenosine monophosphate, cyclic (cAMP)	Energy metabolism and related	HILIC	ESI positive	330.1	136.1	CHEBI:27844	2',3'-cyclic AMP
Carnitine	Energy metabolism and related	HILIC	ESI positive	162.1	103	CHEBI:3424	carnitinium
Glycerol-3-phosphate	Energy metabolism and related	RP-HPLC	ESI negative	170.9	78.8	CHEBI:15978	sn-glycerol 3-phosphate
Hexadecanoylcarnitine	Energy metabolism and related	RP-HPLC	ESI positive	400.2	85	CHEBI:73067	O-palmitoylcarnitine
Hexadecenoylcarnitine	Energy metabolism and related	RP-HPLC	ESI positive	398.2	85	CHEBI:88544	9-Hexadecenoylcarnitine
Hexanoylcarnitine	Energy metabolism and related	HILIC	ESI positive	260.1	85	CHEBI:70749	O-hexanoylcarnitine
O-Acetylcarnitine	Energy metabolism and related	HILIC	ESI positive	204.1	85.2	CHEBI:57589	O-acetyl-L-carnitine
Octadecenoylcarnitine	Energy metabolism and related	RP-HPLC	ESI positive	426.2	85	CHEBI:85460	O-octadecenoyl-L-carnitine
Propionylcarnitine	Energy metabolism and related	HILIC	ESI positive	218.1	85.2	CHEBI:53210	O-propanoyl-L-carnitine
Tetradecanoylcarnitine	Energy metabolism and related	RP-HPLC	ESI positive	372.2	85	CHEBI:73061	O-tetradecanoylcarnitine
2'-Deoxycytidine	Nucleobases and related	HILIC	ESI positive	228.1	112.1	CHEBI:15698	2'-deoxycytidine
Guanine	Nucleobases and related	HILIC	ESI positive	151.9	135.1	CHEBI:16235	guanine

Uridine	Nucleobases and related	HILIC	ESI positive	245.1	113.1	CHEBI:16704	uridine
Uridine	Nucleobases and related	HILIC	ESI negative	243	109.9	CHEBI:16704	uridine
Coenzyme Q10	Vitamins, cofactors and related	RP-HPLC	ESI positive	863.7	197	CHEBI:46245	coenzyme Q10
Coenzyme Q9	Vitamins, cofactors and related	RP-HPLC	ESI positive	795.7	197	CHEBI:18160	ubiquinone-9
Flavin adenine dinucleotide (FAD)	Vitamins, cofactors and related	HILIC	ESI negative	784.4	437.1	CHEBI:16238	FAD
Glutathione (GSH)	Vitamins, cofactors and related	HILIC	ESI negative	306	143	CHEBI:16856	glutathione
Nicotinamide adenine dinucleotide (NAD)	Vitamins, cofactors and related	RP-HPLC	ESI negative	662	540.1	CHEBI:57540	NAD(1−)
Pantothenic acid	Vitamins, cofactors and related	HILIC	ESI negative	218.2	88.1	CHEBI:46905	(R)-pantothenic acid
Pyridoxal	Vitamins, cofactors and related	HILIC	ESI positive	167.9	150	CHEBI:17310	pyridoxal
Thiamine	Vitamins, cofactors and related	HILIC	ESI positive	265.3	122	CHEBI:18385	thiamine(1+)
Triacylglycerol (C30:0,C18:1)	Acylglycerols	RP-HPLC	ESI positive	822.8	523.5	CHEBI:85726	triacylglycerol 48:1
Triacylglycerol (C30:0,C18:2)	Acylglycerols	RP-HPLC	ESI positive	820.8	523.5	CHEBI:85725	triacylglycerol 48:2
Triacylglycerol (C32:0,C16:0)	Acylglycerols	RP-HPLC	ESI positive	824.8	551.5	CHEBI:85870	triacylglycerol 48:0
Triacylglycerol (C32:0,C16:1)	Acylglycerols	RP-HPLC	ESI positive	822.8	551.5	CHEBI:85726	triacylglycerol 48:1
Triacylglycerol (C32:1,C16:1)	Acylglycerols	RP-HPLC	ESI positive	820.8	549.5	CHEBI:85725	triacylglycerol 48:2
Triacylglycerol (C34:0,C16:0)	Acylglycerols	RP-HPLC	ESI positive	852.9	579.6	CHEBI:85874	triacylglycerol 50:0
Triacylglycerol (C34:0,C17:0)	Acylglycerols	RP-HPLC	ESI positive	866.8	579.6	CHEBI:140862	triacylglycerol 51:0
Triacylglycerol (C34:0,C18:0)	Acylglycerols	RP-HPLC	ESI positive	880.9	579.6	CHEBI:167005	triacylglycerol 52:0
Triacylglycerol (C34:0,C18:1)	Acylglycerols	RP-HPLC	ESI positive	878.9	579.6	CHEBI:90302	triacylglycerol 52:1
Triacylglycerol (C34:1,C16:0)	Acylglycerols	RP-HPLC	ESI positive	850.8	577.5	CHEBI:84665	triacylglycerol 50:1
Triacylglycerol (C34:1,C18:1)	Acylglycerols	RP-HPLC	ESI positive	876.9	577.5	CHEBI:85736	triacylglycerol 52:2
Triacylglycerol (C34:1,C18:3)	Acylglycerols	RP-HPLC	ESI positive	872.8	577.5	CHEBI:84660	triacylglycerol 52:4
Triacylglycerol (C34:2,C18:0)	Acylglycerols	RP-HPLC	ESI positive	876.9	575.6	CHEBI:85736	triacylglycerol 52:2
Triacylglycerol (C34:2,C18:1)	Acylglycerols	RP-HPLC	ESI positive	874.9	575.6	CHEBI:84661	triacylglycerol 52:3
Triacylglycerol (C36:1,C18:0)	Acylglycerols	RP-HPLC	ESI positive	906.9	605.4	CHEBI:90305	triacylglycerol 54:1
Triacylglycerol (C36:1,C18:1)	Acylglycerols	RP-HPLC	ESI positive	904.8	605.4	CHEBI:85743	triacylglycerol 54:2
Triacylglycerol (C36:1,C18:2)	Acylglycerols	RP-HPLC	ESI positive	902.9	605.6	CHEBI:84659	triacylglycerol 54:3
Triacylglycerol (C36:2,C18:1)	Acylglycerols	RP-HPLC	ESI positive	902.9	603.6	CHEBI:84659	triacylglycerol 54:3
Triacylglycerol (C36:3,C18:1)	Acylglycerols	RP-HPLC	ESI positive	900.8	601.5	CHEBI:85742	triacylglycerol 54:4
Triacylglycerol (C36:3,C18:2)	Acylglycerols	RP-HPLC	ESI positive	898.8	601.5	CHEBI:85741	triacylglycerol 54:5
Triacylglycerol (C36:4,C16:0)	Acylglycerols	RP-HPLC	ESI positive	872.8	599.5	CHEBI:84660	triacylglycerol 52:4
Triacylglycerol (C36:4,C18:0)	Acylglycerols	RP-HPLC	ESI positive	900.8	599.5	CHEBI:85742	triacylglycerol 54:4
Choline plasmalogen (C36:4)	Glycerophospholipids	RP-HPLC	ESI positive	768.8	184.1	new	phosphatidylcholine P-36:3
Choline plasmalogen (C36:5)	Glycerophospholipids	RP-HPLC	ESI positive	766.8	184.1	CHEBI:132586	phosphatidylcholine P-36:4
Phosphatidylcholine (C32:0)	Glycerophospholipids	RP-HPLC	ESI positive	734.6	184.1	CHEBI:66850	phosphatidylcholine 32:0

Phosphatidylcholine (C34:0)	Glycerophospholipids	RP-HPLC	ESI positive	762.6	184.1	CHEBI:66855	phosphatidylcholine 34:0
Phosphatidylcholine (C34:1)	Glycerophospholipids	RP-HPLC	ESI positive	760.6	184.1	CHEBI:64517	phosphatidylcholine 34:1
Phosphatidylcholine (C34:2)	Glycerophospholipids	RP-HPLC	ESI positive	758.6	184.1	CHEBI:64516	phosphatidylcholine 34:2
Phosphatidylcholine (C34:3)	Glycerophospholipids	RP-HPLC	ESI positive	756.6	184.1	CHEBI:64424	phosphatidylcholine 34:3
Phosphatidylcholine (C36:0)	Glycerophospholipids	RP-HPLC	ESI positive	790.6	184.1	CHEBI:66858	phosphatidylcholine 36:0
Phosphatidylcholine (C36:1)	Glycerophospholipids	RP-HPLC	ESI positive	788.6	184.1	CHEBI:66857	phosphatidylcholine 36:1
Phosphatidylcholine (C36:2)	Glycerophospholipids	RP-HPLC	ESI positive	786.6	184.1	CHEBI:64433	phosphatidylcholine 36:2
Phosphatidylcholine (C36:3)	Glycerophospholipids	RP-HPLC	ESI positive	784.6	184.1	CHEBI:64523	phosphatidylcholine 36:3
Phosphatidylcholine (C36:4)	Glycerophospholipids	RP-HPLC	ESI positive	782.6	184.1	CHEBI:64520	phosphatidylcholine 36:4
Phosphatidylcholine (C36:5)	Glycerophospholipids	RP-HPLC	ESI positive	780.6	184.1	CHEBI:64504	phosphatidylcholine 36:5
Phosphatidylcholine (C38:4)	Glycerophospholipids	RP-HPLC	ESI positive	810.6	184.1	CHEBI:64526	phosphatidylcholine 38:4
Phosphatidylcholine (C38:5)	Glycerophospholipids	RP-HPLC	ESI positive	808.6	184.1	CHEBI:64525	phosphatidylcholine 38:5
Phosphatidylcholine (C38:6)	Glycerophospholipids	RP-HPLC	ESI positive	806.6	184.1	CHEBI:64519	phosphatidylcholine 38:6
Phosphatidylcholine (C40:6)	Glycerophospholipids	RP-HPLC	ESI positive	834.6	184.1	CHEBI:64431	phosphatidylcholine 40:6
Phosphatidylcholine (C40:7)	Glycerophospholipids	RP-HPLC	ESI positive	832.6	184.1	CHEBI:64521	phosphatidylcholine 40:7
Phosphatidylcholine (C40:8)	Glycerophospholipids	RP-HPLC	ESI positive	830.6	184.1	CHEBI:85851	phosphatidylcholine 40:8
Phosphatidylethanolamine (C32:0)	Glycerophospholipids	RP-HPLC	ESI positive	692.6	551.6	CHEBI:71711	phosphatidylethanolamine 32:0
Phosphatidylethanolamine (C34:0)	Glycerophospholipids	RP-HPLC	ESI positive	720.6	579.6	CHEBI:71718	phosphatidylethanolamine 34:0 zwitterion
Phosphatidylethanolamine (C34:1)	Glycerophospholipids	RP-HPLC	ESI positive	718.6	577.6	CHEBI:71720	phosphatidylethanolamine 34:1 zwitterion
Phosphatidylethanolamine (C34:2)	Glycerophospholipids	RP-HPLC	ESI positive	716.5	575.6	CHEBI:71721	phosphatidylethanolamine 34:2 zwitterion
Phosphatidylethanolamine (C36:0)	Glycerophospholipids	RP-HPLC	ESI positive	748.6	607.6	CHEBI:134435	phosphatidylethanolamine 36:0
Phosphatidylethanolamine (C36:1)	Glycerophospholipids	RP-HPLC	ESI positive	746.6	605.6	CHEBI:134451	phosphatidylethanolamine 36:1
Phosphatidylethanolamine (C36:2)	Glycerophospholipids	RP-HPLC	ESI positive	744.6	603.6	CHEBI:141588	phosphatidylethanolamine 36:2
Phosphatidylethanolamine (C36:3)	Glycerophospholipids	RP-HPLC	ESI positive	742.6	601.6	CHEBI:141589	phosphatidylethanolamine 36:3
Phosphatidylethanolamine (C36:4)	Glycerophospholipids	RP-HPLC	ESI positive	740.5	599.6	CHEBI:71730	phosphatidylethanolamine 36:4 zwitterion
Phosphatidylethanolamine (C38:3)	Glycerophospholipids	RP-HPLC	ESI positive	770.6	629.6	CHEBI:141590	phosphatidylethanolamine 38:3
Phosphatidylethanolamine (C38:4)	Glycerophospholipids	RP-HPLC	ESI positive	768.6	627.6	CHEBI:134270	phosphatidylethanolamine 38:4
Phosphatidylethanolamine (C38:5)	Glycerophospholipids	RP-HPLC	ESI positive	766.5	625.6	CHEBI:134239	phosphatidylethanolamine 38:5
Phosphatidylethanolamine (C38:6)	Glycerophospholipids	RP-HPLC	ESI positive	764.5	623.6	CHEBI:134428	phosphatidylethanolamine 38:6
Phosphatidylethanolamine (C40:6)	Glycerophospholipids	RP-HPLC	ESI positive	792.6	651.6	CHEBI:134241	phosphatidylethanolamine 40:6
Phosphatidylethanolamine (C40:7)	Glycerophospholipids	RP-HPLC	ESI positive	790.6	649.6	CHEBI:134429	phosphatidylethanolamine 40:7

Lysophosphatidylcholine (C14:0)	Lysoglycerophospholipids	RP-HPLC	ESI positive	468.3	184.1	CHEBI:64483	lysophosphatidylcholine 14:0
Lysophosphatidylcholine (C16:0)	Lysoglycerophospholipids	RP-HPLC	ESI positive	496.3	184.1	CHEBI:64563	lysophosphatidylcholine 16:0
Lysophosphatidylcholine (C16:1)	Lysoglycerophospholipids	RP-HPLC	ESI positive	494.4	184.1	CHEBI:64560	lysophosphatidylcholine 16:1
Lysophosphatidylcholine (C20:0)	Lysoglycerophospholipids	RP-HPLC	ESI positive	552.4	184.1	CHEBI:67058	lysophosphatidylcholine 20:0
Lysophosphatidylcholine (C20:1)	Lysoglycerophospholipids	RP-HPLC	ESI positive	550.4	184.1	CHEBI:67057	lysophosphatidylcholine 20:1
Lysophosphatidylcholine (C20:4)	Lysoglycerophospholipids	RP-HPLC	ESI positive	544.4	184.1	CHEBI:64568	lysophosphatidylcholine 20:4
Lysophosphatidylcholine (C22:0)	Lysoglycerophospholipids	RP-HPLC	ESI positive	580.4	184.1	CHEBI:67061	lysophosphatidylcholine 22:0
Lysophosphatidylcholine (C24:0)	Lysoglycerophospholipids	RP-HPLC	ESI positive	608.5	184.1	CHEBI:74470	lysophosphatidylcholine 24:0
Lysophosphatidylcholine (C24:1)	Lysoglycerophospholipids	RP-HPLC	ESI positive	606.5	184.1	CHEBI:74471	lysophosphatidylcholine 24:1
Lysophosphatidylethanolamine (C16:0)	Lysoglycerophospholipids	RP-HPLC	ESI positive	454.3	313.2	CHEBI:90452	lysophosphatidylethanolamine 16:0
Lysophosphatidylethanolamine (C18:0)	Lysoglycerophospholipids	RP-HPLC	ESI positive	482.3	341.2	CHEBI:64576	lysophosphatidylethanolamine 18:0
Lysophosphatidylethanolamine (C18:1)	Lysoglycerophospholipids	RP-HPLC	ESI positive	480.3	339.2	CHEBI:64575	lysophosphatidylethanolamine 18:1
Lysophosphatidylethanolamine (C20:4)	Lysoglycerophospholipids	RP-HPLC	ESI positive	502.3	361.2	CHEBI:64569	lysophosphatidylethanolamine 20:4
Lysophosphatidylethanolamine (C22:6)	Lysoglycerophospholipids	RP-HPLC	ESI positive	526.3	385.2	CHEBI:72734	lysophosphatidylethanolamine 22:6
Ceramide (d16:1,C24:0)	Sphingolipids	RP-HPLC	ESI positive	604.2	236.2	new	ceramide d16:1,24:0
Ceramide (d17:1,C24:0)	Sphingolipids	RP-HPLC	ESI positive	618.6	250.2	new	ceramide d17:1,24:0
Ceramide (d18:1,C16:0)	Sphingolipids	RP-HPLC	ESI positive	520.5	264.2	CHEBI:184082	N-palmitoyl-d-sphingosine
Ceramide (d18:1,C18:0)	Sphingolipids	RP-HPLC	ESI positive	548.5	264.2	new	ceramide d18:1,18:0
Ceramide (d18:1,C20:0)	Sphingolipids	RP-HPLC	ESI positive	576.6	264.2	new	ceramide d18:1,20:0
Ceramide (d18:1,C21:0)	Sphingolipids	RP-HPLC	ESI positive	590.5	264.2	new	ceramide d18:1,21:0
Ceramide (d18:1,C22:0)	Sphingolipids	RP-HPLC	ESI positive	604.6	264.2	new	ceramide d18:1,22:0
Ceramide (d18:1,C22:1)	Sphingolipids	RP-HPLC	ESI positive	602.6	264.2	new	ceramide d18:1,22:1
Ceramide (d18:1,C23:0)	Sphingolipids	RP-HPLC	ESI positive	618.6	264.2	new	ceramide d18:1,23:0
Ceramide (d18:1,C24:0)	Sphingolipids	RP-HPLC	ESI positive	632.6	264.2	new	ceramide d18:1,24:0
Ceramide (d18:1,C24:1)	Sphingolipids	RP-HPLC	ESI positive	630.6	264.2	new	ceramide d18:1,24:1
Ceramide (d18:1,C24:2)	Sphingolipids	RP-HPLC	ESI positive	628.6	264.2	new	ceramide d18:1,24:2
Ceramide (d18:2,C16:0)	Sphingolipids	RP-HPLC	ESI positive	518.5	262.2	new	ceramide d18:2,16:0
Ceramide (d18:2,C18:0)	Sphingolipids	RP-HPLC	ESI positive	546.5	262.2	new	ceramide d18:2,18:0
Ceramide (d18:2,C22:0)	Sphingolipids	RP-HPLC	ESI positive	602.6	262.2	new	ceramide d18:2,22:0
Ceramide (d18:2,C23:0)	Sphingolipids	RP-HPLC	ESI positive	616.5	262.2	new	ceramide d18:2,23:0
Ceramide (d18:2,C24:0)	Sphingolipids	RP-HPLC	ESI positive	630.6	262.2	new	ceramide d18:2,24:0
Ceramide (d18:2,C24:1)	Sphingolipids	RP-HPLC	ESI positive	628.6	262.2	new	ceramide d18:2,24:1

Ceramide (d18:2,C24:2)	Sphingolipids	RP-HPLC	ESI positive	626.6	262.2	new	ceramide d18:2,24:2
Sphingomyelin (d32:1)	Sphingolipids	RP-HPLC	ESI positive	675.5	184.1	CHEBI:64586	sphingomyelin 32:1
Sphingomyelin (d32:2)	Sphingolipids	RP-HPLC	ESI positive	673.5	184.1	CHEBI:72510	sphingomyelin 32:2
Sphingomyelin (d33:1)	Sphingolipids	RP-HPLC	ESI positive	689.6	184.1	CHEBI:64585	sphingomyelin 33:1
Sphingomyelin (d34:0)	Sphingolipids	RP-HPLC	ESI positive	705.5	184.1	CHEBI:72513	sphingomyelin 34:0
Sphingomyelin (d34:1)	Sphingolipids	RP-HPLC	ESI positive	703.6	184.1	CHEBI:72514	sphingomyelin 34:1
Sphingomyelin (d34:1)	Sphingolipids	RP-HPLC	ESI positive	725.6	542.5	CHEBI:72514	sphingomyelin 34:1
Sphingomyelin (d34:2)	Sphingolipids	RP-HPLC	ESI positive	701.5	184.1	CHEBI:64587	sphingomyelin 34:2
Sphingomyelin (d34:2)	Sphingolipids	RP-HPLC	ESI positive	723.6	540.6	CHEBI:64587	sphingomyelin 34:2
Sphingomyelin (d35:1)	Sphingolipids	RP-HPLC	ESI positive	717.5	184.1	CHEBI:133629	sphingomyelin 35:1
Sphingomyelin (d35:2)	Sphingolipids	RP-HPLC	ESI positive	715.5	184.1	new	sphingomyelin 35:2
Sphingomyelin (d36:1)	Sphingolipids	RP-HPLC	ESI positive	731.5	184.1	CHEBI:72518	sphingomyelin 36:1
Sphingomyelin (d36:2)	Sphingolipids	RP-HPLC	ESI positive	729.5	184.1	CHEBI:72519	sphingomyelin 36:2
Sphingomyelin (d36:3)	Sphingolipids	RP-HPLC	ESI positive	727.5	184.1	CHEBI:72520	sphingomyelin 36:3
Sphingomyelin (d37:1)	Sphingolipids	RP-HPLC	ESI positive	745.5	184.1	CHEBI:85759	sphingomyelin 37:1
Sphingomyelin (d38:1)	Sphingolipids	RP-HPLC	ESI positive	759.5	184.1	CHEBI:72523	sphingomyelin 38:1
Sphingomyelin (d38:2)	Sphingolipids	RP-HPLC	ESI positive	757.5	184.1	CHEBI:72524	sphingomyelin 38:2
Sphingomyelin (d39:1)	Sphingolipids	RP-HPLC	ESI positive	773.5	184.1	CHEBI:85761	sphingomyelin 39:1
Sphingomyelin (d40:1)	Sphingolipids	RP-HPLC	ESI positive	787.5	184.1	CHEBI:72528	sphingomyelin 40:1
Sphingomyelin (d40:2)	Sphingolipids	RP-HPLC	ESI positive	785.5	184.1	CHEBI:72529	sphingomyelin 40:2
Sphingomyelin (d41:1)	Sphingolipids	RP-HPLC	ESI positive	801.5	184.1	CHEBI:83893	sphingomyelin 41:1
Sphingomyelin (d41:2)	Sphingolipids	RP-HPLC	ESI positive	799.7	184.1	CHEBI:85762	sphingomyelin 41:2
Sphingomyelin (d42:1)	Sphingolipids	RP-HPLC	ESI positive	815.7	184.1	CHEBI:72533	sphingomyelin 42:1
Sphingomyelin (d42:2)	Sphingolipids	RP-HPLC	ESI positive	813.7	184.1	CHEBI:72534	sphingomyelin 42:2
Cholesterylester (C20:2)	Cholesterol and related	RP-HPLC	ESI positive	694.7	369.3	CHEBI:183804	CE(20:2)
Cholesterylester (C20:3)	Cholesterol and related	RP-HPLC	ESI positive	692.7	369.3	CHEBI:138331	cholesteryl icosatrienoate
Isopentenyl pyrophosphate (IPP)	Cholesterol and related	RP-HPLC	ESI negative	245	78.8	CHEBI:16584	isopentenyl diphosphate
Phosphocholine	Miscellaneous lipids	HILIC	ESI positive	183.8	86.2	CHEBI:18132	phosphocholine
Biliverdin	Miscellaneous	HILIC	ESI positive	583.3	297.2	CHEBI:17033	biliverdin
Unknown lipid (849590045)	Unknown	RP-HPLC	ESI positive	794.6	184.1	n/a	n/a
Unknown lipid (849590046)	Unknown	RP-HPLC	ESI positive	811.6	183.9	n/a	n/a
Unknown lipid (849590126)	Unknown	RP-HPLC	ESI positive	772.6	184.1	n/a	n/a
Unknown lipid (849590204)	Unknown	RP-HPLC	ESI positive	870.8	597.5	n/a	n/a
Unknown lipid (849590225)	Unknown	RP-HPLC	ESI positive	904	904	n/a	n/a
Unknown lipid (849590328)	Unknown	RP-HPLC	ESI positive	428.1	136.2	n/a	n/a
Unknown lipid (849590410)	Unknown	RP-HPLC	ESI positive	523.5	211.2	n/a	n/a
Unknown lipid (849590418)	Unknown	RP-HPLC	ESI positive	575.5	237.2	n/a	n/a
Unknown lipid (849590419)	Unknown	RP-HPLC	ESI positive	575.5	265.3	n/a	n/a
Unknown lipid (849590423)	Unknown	RP-HPLC	ESI positive	577.5	239.2	n/a	n/a

Unknown lipid (849590430)	Unknown	RP-HPLC	ESI positive	603.5	135.1	n/a	n/a
Unknown lipid (849590434)	Unknown	RP-HPLC	ESI positive	620.6	236.2	n/a	n/a
Unknown lipid (849590435)	Unknown	RP-HPLC	ESI positive	620.6	262.2	n/a	n/a
Unknown lipid (849590436)	Unknown	RP-HPLC	ESI positive	620.6	264.2	n/a	n/a
Unknown lipid (849590437)	Unknown	RP-HPLC	ESI positive	622.6	236.2	n/a	n/a
Unknown lipid (849590438)	Unknown	RP-HPLC	ESI positive	622.6	264.2	n/a	n/a
Unknown lipid (849590439)	Unknown	RP-HPLC	ESI positive	631.6	265.3	n/a	n/a
Unknown lipid (849590442)	Unknown	RP-HPLC	ESI positive	634.6	262.2	n/a	n/a
Unknown lipid (849590443)	Unknown	RP-HPLC	ESI positive	634.6	264.2	n/a	n/a
Unknown lipid (849590444)	Unknown	RP-HPLC	ESI positive	636.6	250.2	n/a	n/a
Unknown lipid (849590445)	Unknown	RP-HPLC	ESI positive	636.6	264.2	n/a	n/a
Unknown lipid (849590446)	Unknown	RP-HPLC	ESI positive	646.6	264.2	n/a	n/a
Unknown lipid (849590448)	Unknown	RP-HPLC	ESI positive	648.6	262.2	n/a	n/a
Unknown lipid (849590449)	Unknown	RP-HPLC	ESI positive	648.6	264.2	n/a	n/a
Unknown lipid (849590450)	Unknown	RP-HPLC	ESI positive	650.6	264.2	n/a	n/a
Unknown lipid (849590451)	Unknown	RP-HPLC	ESI positive	662.6	264.2	n/a	n/a
Unknown lipid (849590452)	Unknown	RP-HPLC	ESI positive	662.6	278.2	n/a	n/a
Unknown lipid (849590454)	Unknown	RP-HPLC	ESI positive	704.5	184.1	n/a	n/a
Unknown lipid (849590455)	Unknown	RP-HPLC	ESI positive	716.5	184.1	n/a	n/a
Unknown lipid (849590456)	Unknown	RP-HPLC	ESI positive	728.5	184.1	n/a	n/a
Unknown lipid (849590457)	Unknown	RP-HPLC	ESI positive	730.5	184.1	n/a	n/a
Unknown lipid (849590458)	Unknown	RP-HPLC	ESI positive	732.5	184.1	n/a	n/a
Unknown lipid (849590459)	Unknown	RP-HPLC	ESI positive	732.6	184.1	n/a	n/a
Unknown lipid (849590460)	Unknown	RP-HPLC	ESI positive	738.5	184.1	n/a	n/a
Unknown lipid (849590462)	Unknown	RP-HPLC	ESI positive	744.6	184.1	n/a	n/a
Unknown lipid (849590463)	Unknown	RP-HPLC	ESI positive	764.5	184.1	n/a	n/a
Unknown lipid (849590464)	Unknown	RP-HPLC	ESI positive	774.6	184.1	n/a	n/a
Unknown lipid (849590465)	Unknown	RP-HPLC	ESI positive	778.5	184.1	n/a	n/a
Unknown lipid (849590466)	Unknown	RP-HPLC	ESI positive	792.6	184.1	n/a	n/a
Unknown lipid (849590467)	Unknown	RP-HPLC	ESI positive	794.6	653.6	n/a	n/a
Unknown lipid (849590468)	Unknown	RP-HPLC	ESI positive	796.6	184.1	n/a	n/a
Unknown lipid (849590469)	Unknown	RP-HPLC	ESI positive	796.6	655.6	n/a	n/a
Unknown lipid (849590472)	Unknown	RP-HPLC	ESI positive	824.6	184.1	n/a	n/a
Unknown lipid (849590473)	Unknown	RP-HPLC	ESI positive	824.6	683.6	n/a	n/a
Unknown lipid (849590480)	Unknown	RP-HPLC	ESI positive	838.6	184.1	n/a	n/a
Unknown lipid (849590481)	Unknown	RP-HPLC	ESI positive	842.6	184.1	n/a	n/a
Unknown lipid (849590482)	Unknown	RP-HPLC	ESI positive	848.8	549.5	n/a	n/a
Unknown lipid (849590483)	Unknown	RP-HPLC	ESI positive	848.8	575.5	n/a	n/a
Unknown lipid (849590484)	Unknown	RP-HPLC	ESI positive	848.8	577.5	n/a	n/a
Unknown lipid (849590485)	Unknown	RP-HPLC	ESI positive	850.8	551.5	n/a	n/a

Unknown lipid (849590486)	Unknown	RP-HPLC	ESI positive	850.8	605.5	n/a	n/a
Unknown polar (869590388)	Unknown	HILIC	ESI positive	102.1	56.1	n/a	n/a
Unknown polar (869590390)	Unknown	HILIC	ESI positive	116.1	70.1	n/a	n/a
Unknown polar (869590398)	Unknown	HILIC	ESI positive	132.1	90.1	n/a	n/a
Unknown polar (869590402)	Unknown	HILIC	ESI positive	148.1	56	n/a	n/a
Unknown polar (869590442)	Unknown	HILIC	ESI positive	230.2	100.1	n/a	n/a
Unknown polar (869590444)	Unknown	HILIC	ESI positive	232.2	85	n/a	n/a
Unknown polar (869590448)	Unknown	HILIC	ESI positive	246.2	85	n/a	n/a
Unknown polar (869590452)	Unknown	HILIC	ESI positive	268.1	136.1	n/a	n/a
Unknown polar (869590453)	Unknown	HILIC	ESI positive	296.1	104.1	n/a	n/a
Unknown polar (869590456)	Unknown	HILIC	ESI positive	298.1	136.1	n/a	n/a
Unknown polar (869590459)	Unknown	HILIC	ESI positive	330.1	285.1	n/a	n/a
Unknown polar (879590076)	Unknown	HILIC	ESI negative	245.1	245.1	n/a	n/a
Unknown polar (879590422)	Unknown	HILIC	ESI negative	308.1	146.1	n/a	n/a
Unknown polar (879590425)	Unknown	HILIC	ESI negative	540.1	273	n/a	n/a

Suppl Fig 2. PCA and loading plots the metabolic profiles of different cell seeding densities and passages before and after biomass normalization. a) PCA and b) loading plots of unnormalized metabolic profiles of different cell seeding densities and passages (5 and7). Normalization for cell number correction was <u>not</u> performed. c)PCA and c) loading plots of Sample Analyte Median (SAM) normalized metabolic profiles of different cell seeding densities and passages and passages (5 and7).

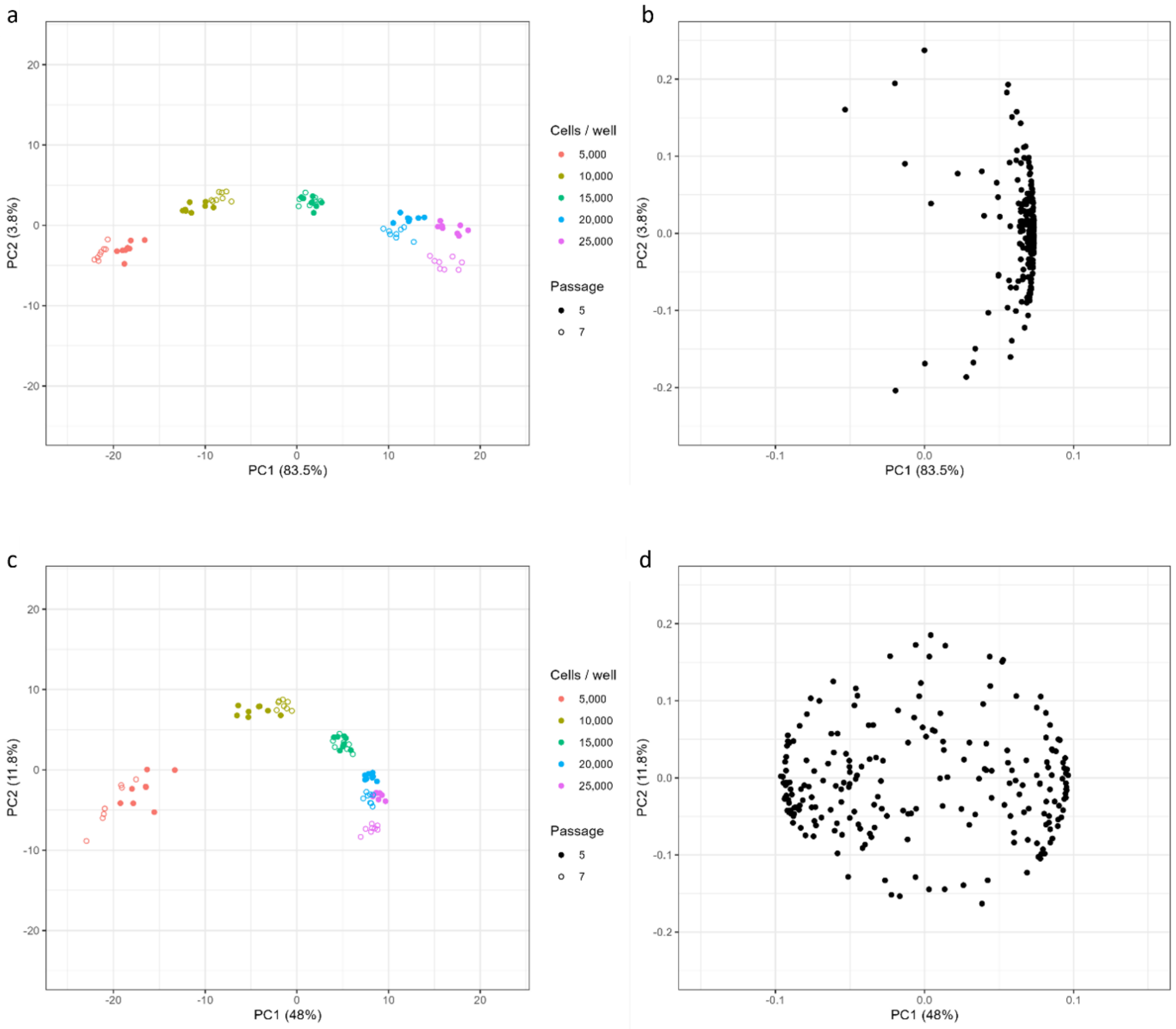

Suppl Fig. 3 Cytotoxicity and cell viability range finder for dose selection. Cell viability assay (CellTiter-Glo®) and Cytotoxicity assay (CellTox™ Green) n=6.a) Acifluorfen, b) Wy-14643, c) β Naphthoflavone, d) Aroclor 1254, e) Pendimethalin, f) Ketoconazole. Values are presented as percentage of vehicle controls for CellTiter-Glo® and as percentage of positive control (lysis buffer) for CellTox™ Green.

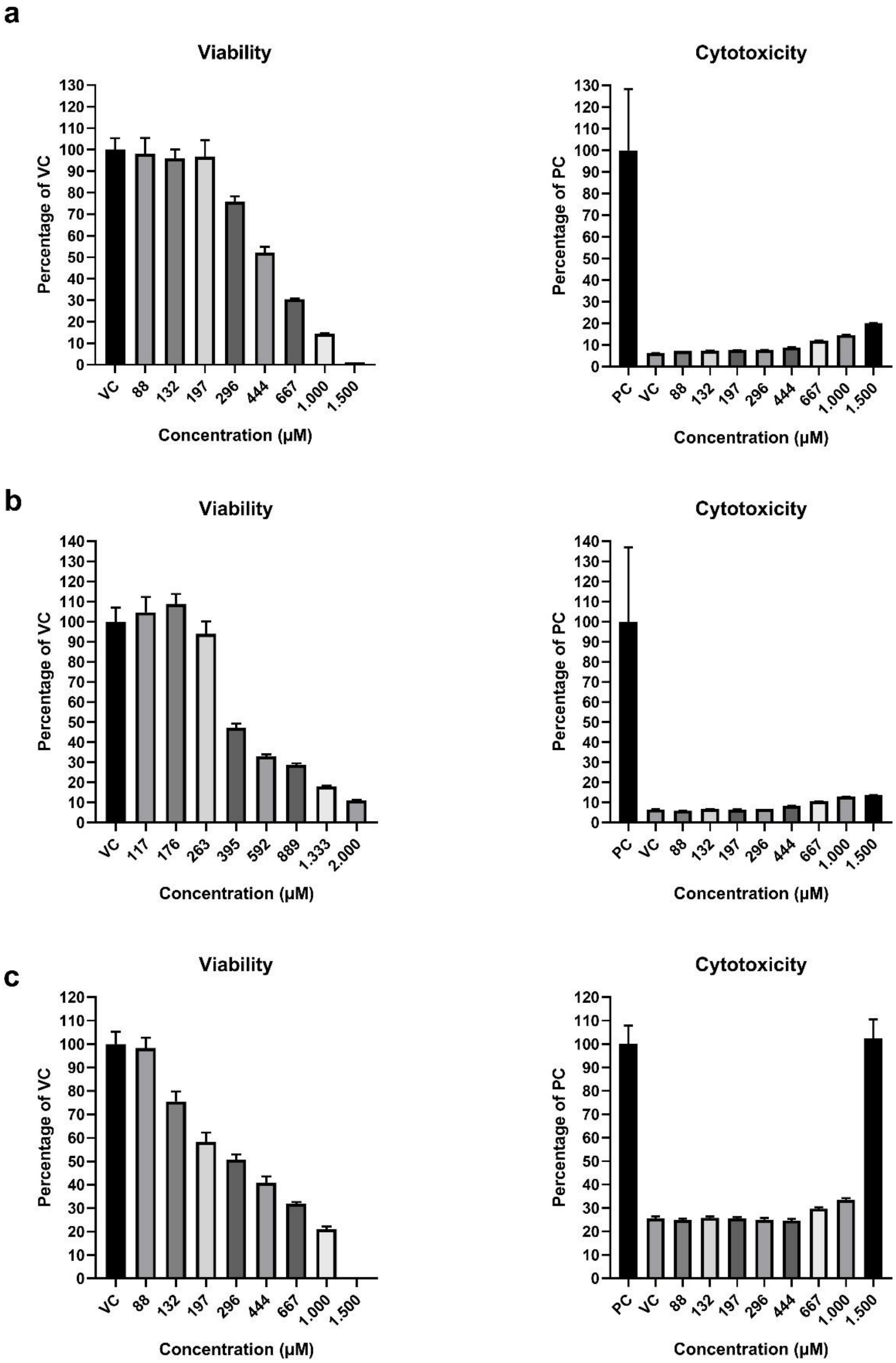

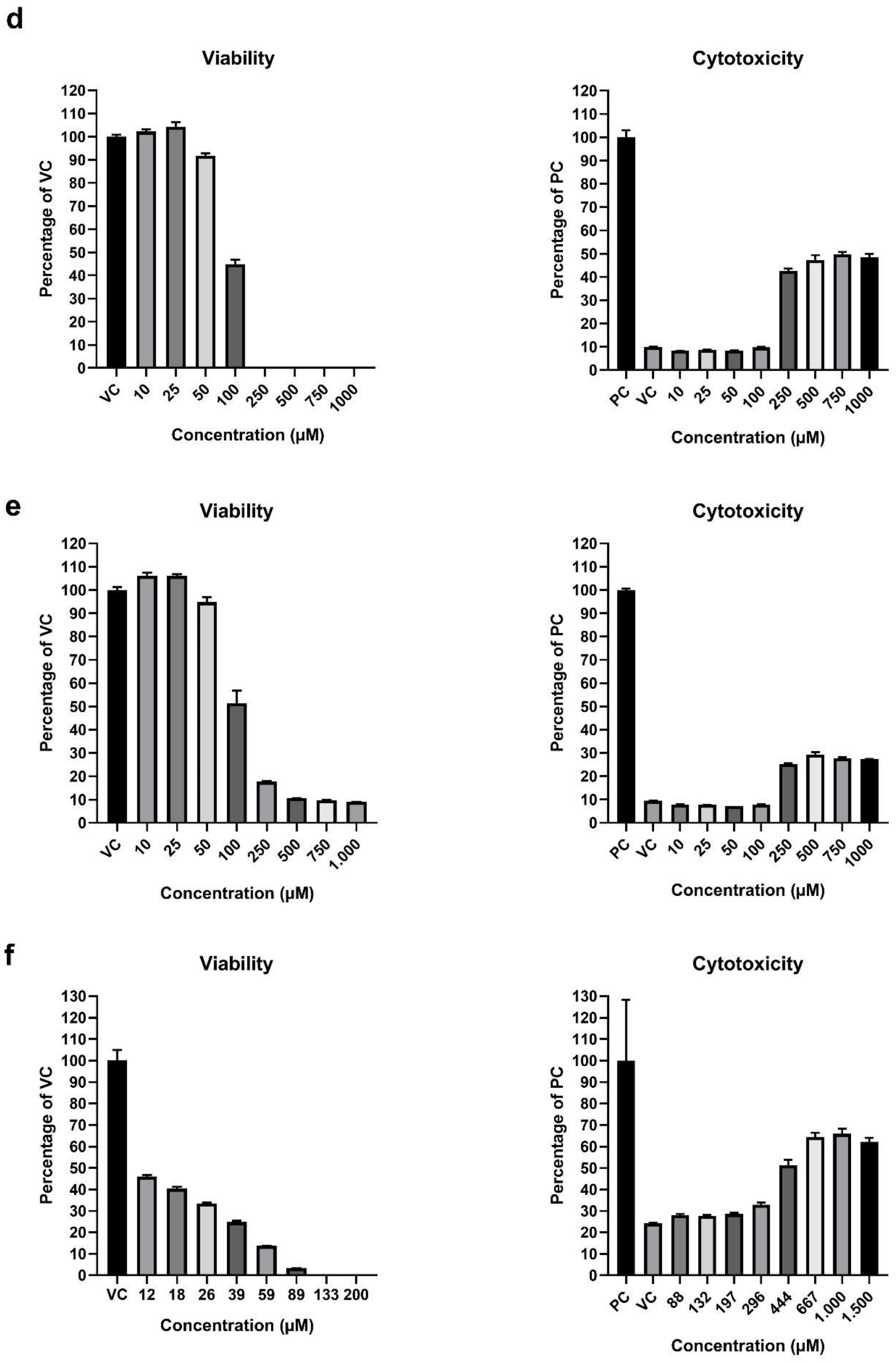
d
Viability
Percentage of VC
0
10
20
30
40
50
60
70
80
90
100
110
120
VC
10
25
50
100
250
500
750
1000
Concentration (µM)
Cytotoxicity
Percentage of PC
0
10
20
30
40
50
60
70
80
90
100
110
120
PC
VC
10
25
50
100
250
500
750
1000
Concentration (µM)
e
Viability
Percentage of VC
0
10
20
30
40
50
60
70
80
90
100
110
120
VC
10
25
50
100
250
500
750
1.000
Concentration (µM)
Cytotoxicity
Percentage of PC
0
10
20
30
40
50
60
70
80
90
100
110
120
PC
VC
10
25
50
100
250
500
750
1000
Concentration (µM)
f
Viability
Percentage of VC
0
10
20
30
40
50
60
70
80
90
100
110
120
130
VC
12
18
26
39
59
89
133
200
Concentration (µM)
Cytotoxicity
Percentage of PC
0
10
20
30
40
50
60
70
80
90
100
110
120
130
PC
VC
88
132
197
296
444
667
1.000
1.500
Concentration (µM)

Suppl Fig. 4 Dose response curves for dose selection. Dose response curves were built with the ATP measurement (CellTiter-Glo®) in the range finder experiments (see Suppl. Figure 2) and used to derive effective concentration (EC) for the metabolomics dose setting. Acifluorfen, b) Wy-14643, c) β-Naphthoflavone, d) Aroclor 1254, e) Pendimethalin, f) Ketoconazole.

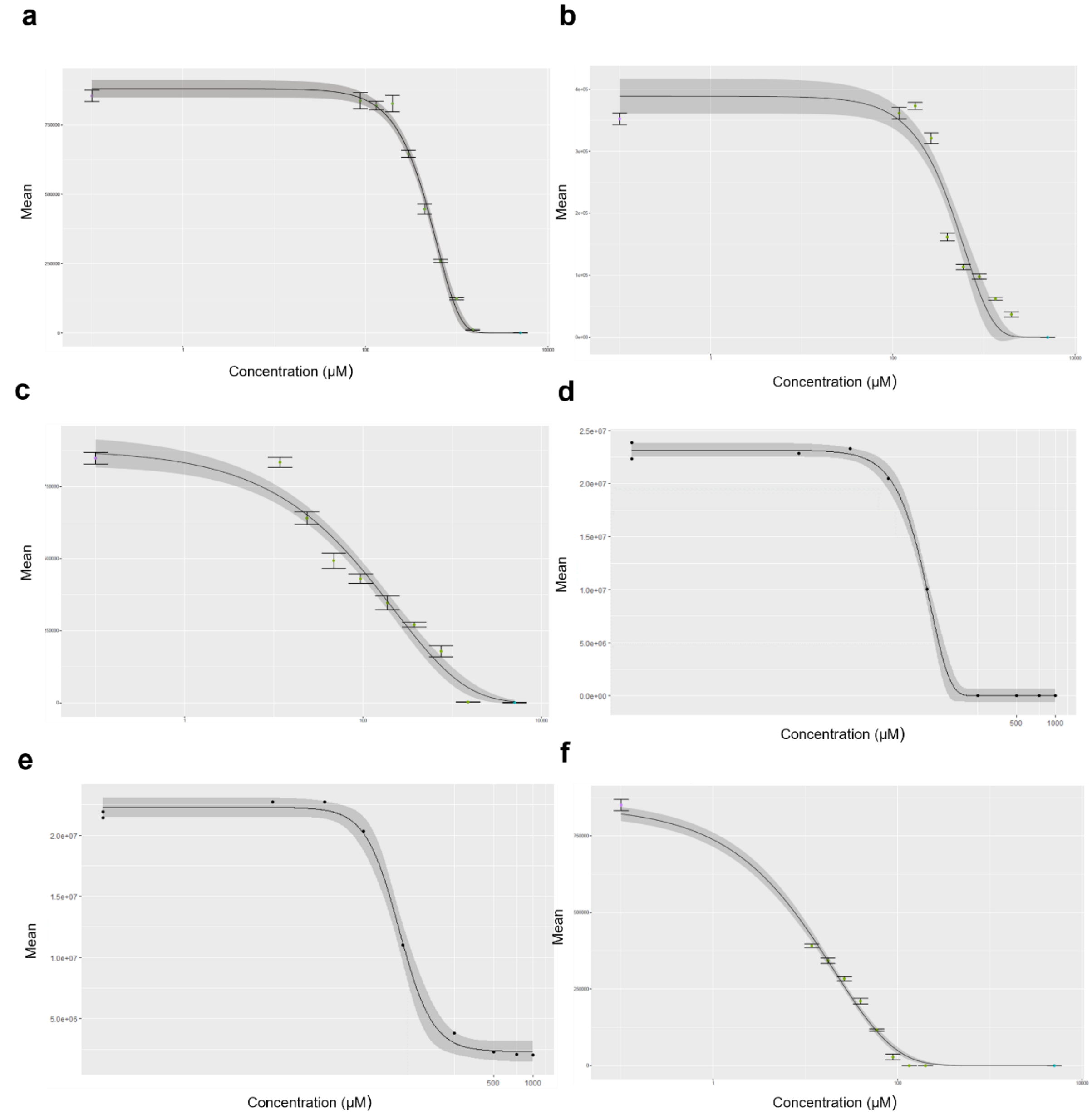

Suppl Fig. 5 *Metabolomics experiment cell viability and cytotoxicity testing. Cell viability assay (CellTiter-Glo®) and Cytotoxicity assay (CellTox™ Green) n=6 were carried out in parallel with metabolomics experiments in plates handled and treated exactly as the ones used for metabolite profiling. a) Acifluorfen, b) Wy-14643, c) β-Naphthoflavone, d) Aroclor 1254, e) Pendimethalin, f) Ketoconazole.*

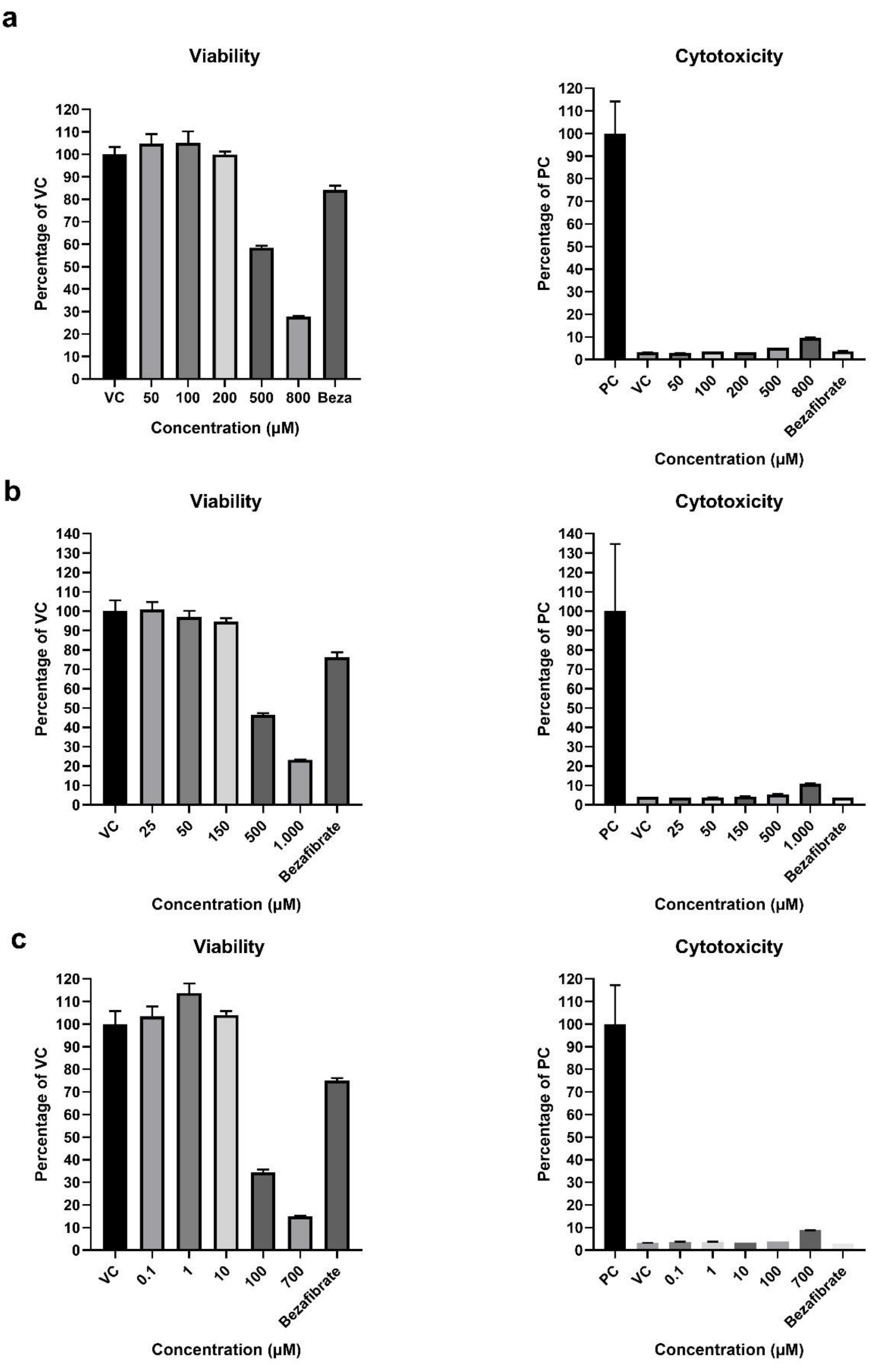

d

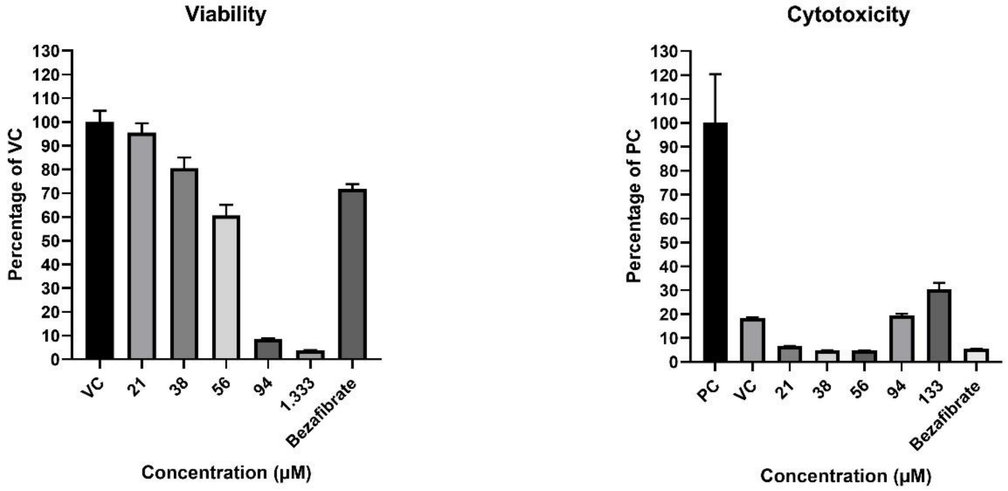
Viability
Percentage of VC
130
120
110
100
90
80
70
60
50
40
30
20
10
0
VC
21
38
56
94
1.333
Bezafibrate
Concentration (µM)
Cytotoxicity
Percentage of PC
130
120
110
100
90
80
70
60
50
40
30
20
10
0
PC
VC
21
38
56
94
133
Bezafibrate
Concentration (µM)

e

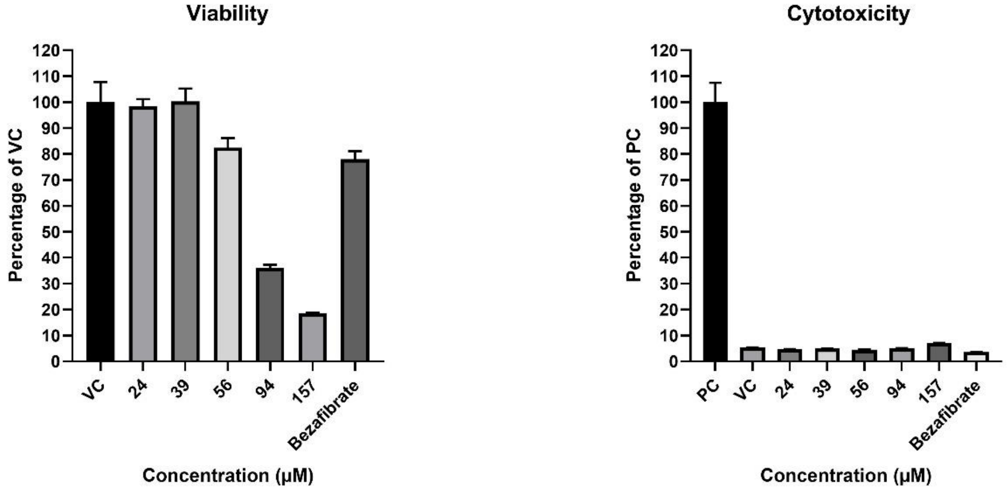
Viability
Percentage of VC
120
110
100
90
80
70
60
50
40
30
20
10
0
VC
24
39
56
94
157
Bezafibrate
Concentration (µM)
Cytotoxicity
Percentage of PC
120
110
100
90
80
70
60
50
40
30
20
10
0
PC
VC
24
39
56
94
157
Bezafibrate
Concentration (µM)

f

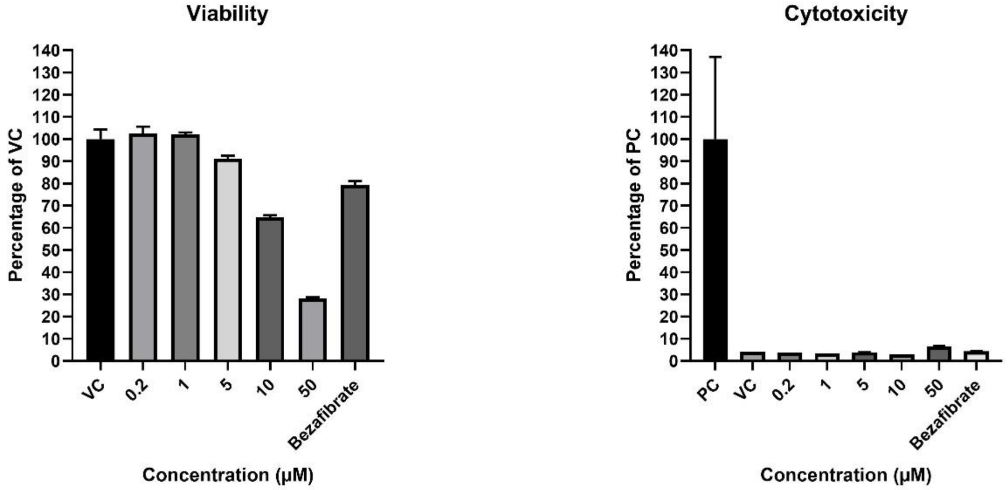
Viability
Percentage of VC
140
130
120
110
100
90
80
70
60
50
40
30
20
10
0
VC
0.2
1
5
10
50
Bezafibrate
Concentration (µM)
Cytotoxicity
Percentage of PC
140
130
120
110
100
90
80
70
60
50
40
30
20
10
0
PC
VC
0.2
1
5
10
50
Bezafibrate
Concentration (µM)

Suppl Fig 6. *Enrichment analysis of significantly altered metabolites by ontology class after substance treatment. The distribution of the 241measured metabolites across the ontology classes is provided in the column "measured metabolites". The number of metabolite changes are shown for each metabolite ontology class. Numbers yellow represent that a treatment caused a significant (p-value<0.05) enrichment in an ontology class. C1 to C5: substance concentrations. Acifluorfen, b) Wy-14643, c) β-Naphthoflavone, d) Aroclor 1254, e) Pendimethalin, f) Ketoconazole.*

	Substance	Acifluorfen				
	Concentration	C1	C2	C3	C4	C5
Metabolite Class	Measured metabolites	UP	UP	UP	UP	UP
Acylglycerols	17	14	16	17	16	15
Amino acids	13	1	1	1	5	8
Amino acids related	9	1	1	1	3	3
Cholesterol and related	3	0	0	0	1	2
Energy metabolism and related	9	1	1	0	2	3
Glycerophospholipids	32	2	4	3	7	10
Hormones, signal substances and related	2	0	0	0	0	0
Lysoglycerophospholipids	29	4	8	10	23	24
Miscellaneous	1	0	0	1	1	0
Nucleobases and related	4	0	0	0	0	0
Sphingolipids	37	4	3	3	4	7
Unknown	76	17	18	22	29	31
Vitamins, cofactors and related	9	1	0	0	1	1
Total # Metabolites	241	45	52	58	92	104

	Substance	Wyth				
	Concentration	C1	C2	C3	C4	C5
Metabolite Class	Measured metabolites					
Acylglycerols	17	0	4	4	10	10
Amino acids	13	0	0	2	1	1
Amino acids related	9	0	0	1	3	3
Cholesterol and related	3	0	0	0	0	1
Energy metabolism and related	9	0	0	1	4	3
Glycerophospholipids	32	1	1	9	8	17
Hormones, signal substances and related	2	0	0	0	2	2
Lysoglycerophospholipids	29	0	1	1	6	6
Miscellaneous	1	0	0	0	0	0
Nucleobases and related	4	0	0	1	1	0
Sphingolipids	37	2	3	12	17	17
Unknown	76	1	4	19	35	40
Vitamins, cofactors and related	9	0	1	3	2	4
Total # Metabolites	241	4	14	53	89	104

	Substance	Aroclor				
	Concentration	C1	C2	C3	C4	C5
Metabolite Class	Measured metabolites					
Acylglycerols	17	1	0	6	10	8
Amino acids	13	0	0	7	11	11
Amino acids related	9	0	2	3	5	4
Cholesterol and related	3	0	0	0	2	2
Energy metabolism and related	9	0	0	4	5	5
Glycerophospholipids	32	10	13	11	9	8
Hormones, signal substances and related	2	0	0	0	0	0
Lysoglycerophospholipids	29	13	15	20	17	18
Miscellaneous	1	0	0	0	0	0
Nucleobases and related	4	0	0	1	3	4
Sphingolipids	37	6	16	11	8	13
Unknown	76	5	14	17	24	26
Vitamins, cofactors and related	9	1	4	1	3	3
Total # Metabolites	241	36	64	81	97	102

	Substance	Pendimethalin				
	Concentration	C1	C2	C3	C4	C5
Metabolite Class	Measured metabolites					
Acylglycerols	17	3	4	4	7	8
Amino acids	13	0	0	1	10	11
Amino acids related	9	1	1	1	2	3
Cholesterol and related	3	0	0	0	0	2
Energy metabolism and related	9	0	0	0	2	5
Glycerophospholipids	32	7	7	8	13	10
Hormones, signal substances and related	2	0	0	0	0	0
Lysoglycerophospholipids	29	21	22	25	24	21
Miscellaneous	1	0	0	0	0	0
Nucleobases and related	4	0	0	1	1	0
Sphingolipids	37	4	5	9	15	18
Unknown	76	5	7	12	18	17
Vitamins, cofactors and related	9	0	1	1	2	2
Total # Metabolites	241	41	47	62	94	97

	Substance	ß-Naphthoflavon plate				
	Concentration	C1	C2	C3	C4	C5
Metabolite Class	Measured metabolites					
Acylglycerols	17	0	0	0	0	0
Amino acids	13	0	0	0	4	0
Amino acids related	9	0	0	0	0	0
Cholesterol and related	3	0	0	0	0	1
Energy metabolism and related	9	0	0	3	2	2
Glycerophospholipids	32	4	11	16	15	13
Hormones, signal substances and related	2	0	0	0	0	0
Lysoglycerophospholipids	29	15	19	25	27	26
Miscellaneous	1	0	0	1	1	1
Nucleobases and related	4	0	0	0	1	1
Sphingolipids	37	4	4	6	7	6
Unknown	76	4	9	14	13	13
Vitamins, cofactors and related	9	1	2	4	4	2
Total # Metabolites	241	28	45	69	74	65

	Substance	Ketoconazole				
	Concentration	C1	C2	C3	C4	C5
Metabolite Class	Measured metabolites					
Acylglycerols	17	0	8	9	9	11
Amino acids	13	4	3	6	7	2
Amino acids related	9	3	1	5	8	5
Cholesterol and related	3	0	0	2	1	0
Energy metabolism and related	9	0	0	1	4	4
Glycerophospholipids	32	2	10	19	22	25
Hormones, signal substances and related	2	0	0	0	2	2
Lysoglycerophospholipids	29	1	2	1	2	0
Miscellaneous	1	0	0	0	0	1
Nucleobases and related	4	0	0	0	1	1
Sphingolipids	37	1	6	13	14	6
Unknown	76	10	17	44	51	51
Vitamins, cofactors and related	9	2	0	2	5	5
Total # Metabolites	241	23	47	102	126	113

Suppl Fig 7*. Experimental variability and reproducibility. the variance of every log-transformed metabolite for both pooled samples (technical replicates) and control samples was calculated. These variances were back transformed to linear scale, yielding a relative standard deviation (RSD).*

Plate	RSD Pool	RSD Controls
Acifluorfen	0.10	0.11
Aroclor 1254	0.10	0.11
Ketoconazole	0.09	0.09
Naphthoflavone	0.10	0.09
Pendimethalin	0.08	0.11
Wy-14643	0.10	0.10

Suppl Fig. 8 *3D-PCA of metabolic profiles shows a MoA-specific clustering of liver toxicants**.** PCA of metabolite profiles of HepG2 cells treated for 48h with three liver enzyme inducers (Pendimethalin, Aroclor, β-Naphthoflavone), three peroxisome proliferators (Bezafibrate, Acifluorfen, Wy-14643) and one liver enzyme inhibitor (Ketoconazole) allows to discriminate between the different mode of actions of these substances.*

Intermediate concentrations (C3 for Pendimethalin, B-naphthoflavone, acifluorfen, Wy-14643, and Ketoconazole and C2 for Aroclor) were selected for the analysis.

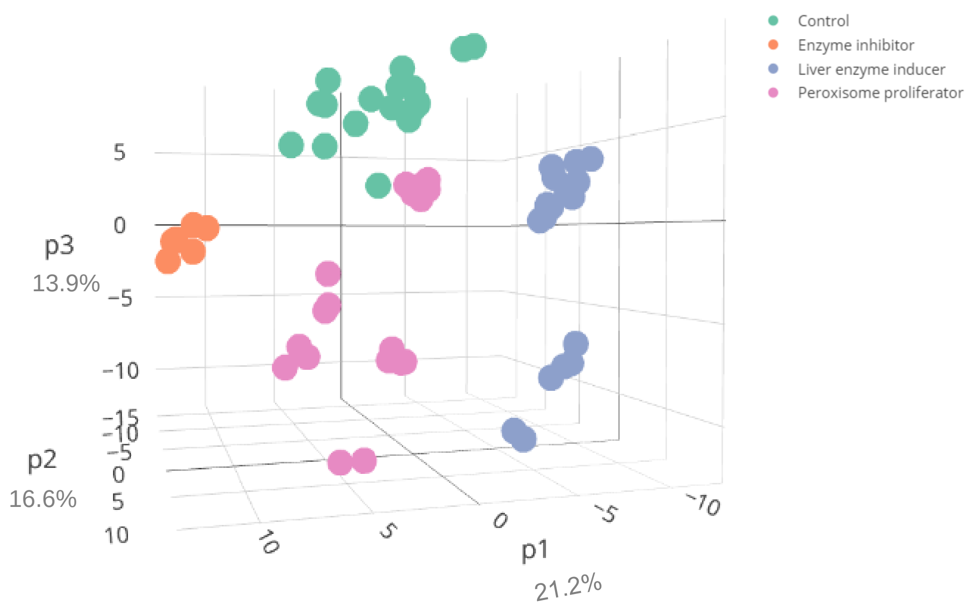

Suppl Fig. 9 Metabolome changes induced by peroxisome proliferators. *Heatmap of common statistically significantly (p<0.05) altered metabolites after Bezafibrate, Acifluorfen and Wy -14643 treatment. Red represents significantly upregulated metabolites and blue represents significantly downregulated metabolites as compared to controls. C3 equivalent to EC15ATP was used for the analysis. Bezafibrate was used as a positive control in each of the 6 plates of the experiment (n=6 per condition).*

Metabolite	Bezafibrate Plate 1	Bezafibrate Plate 2	Bezafibrate Plate 3	Bezafibrate Plate 4	Bezafibrate Plate 5	Bezafibrate Plate 6	Wy-14643	Acifluorfen	Metabolite class
Taurine	0.93	0.92	0.68	0.93	0.90	0.88	0.97	0.61	Amino acids
5-Hydroxytryptophan	0.85	0.74	0.65	0.84	0.77	0.76	0.85	0.74	Amino acids related
Creatine	0.80	0.82	0.73	0.78	0.67	0.66	0.85	0.87	
Ketoleucine	0.73	0.72	0.74	0.73	0.77	1.10	0.86	0.80	
N-Acetylaspartate	1.54	1.49	1.66	1.48	1.41	1.25	1.22	1.50	
S-Adenosylhomocysteine	0.92	0.98	0.70	0.93	0.78	0.79	1.03	0.84	
Carnitine	0.72	0.74	0.84	0.72	0.77	0.80	0.99	0.98	Energy metabolism and related
Glycerol-3-phosphate	0.68	0.43	0.45	0.54	0.44	0.39	0.72	0.90	
Hexanoylcarnitine	0.65	0.54	0.50	0.59	0.59	0.62	0.93	0.70	
Propionylcarnitine	0.66	0.78	0.40	0.58	0.54	0.57	0.97	0.79	
2'-Deoxycytidine	0.37	0.42	0.16	0.27	0.22	0.21	0.75	0.56	Nucleobases and related
Pantothenic acid	0.68	0.77	0.59	0.72	0.70	0.76	0.74	0.68	Vitamins. cofactors and related
Triacylglycerol (C34:1.C18:3)	1.57	2.06	2.37	1.58	1.93	2.00	1.01	1.73	Acylglycerols
Triacylglycerol (C34:2.C18:0)	1.63	1.54	1.78	1.17	1.31	1.39	1.14	2.66	
Triacylglycerol (C34:2.C18:1)	1.40	1.62	1.81	1.26	1.79	1.43	1.01	2.04	
Triacylglycerol (C36:4.C16:0)	1.74	2.34	2.73	1.73	1.97	2.11	1.09	1.86	
Triacylglycerol (C36:4.C18:0)	2.15	2.38	2.26	1.72	1.82	2.28	1.18	2.14	
Choline plasmalogen (C36:4)	0.65	0.80	0.68	0.69	0.73	0.72	1.00	0.75	Glycerophospholipids
Choline plasmalogen (C36:5)	0.78	0.90	0.80	0.83	0.82	0.79	1.03	0.70	
Phosphatidylcholine (C32:0)	0.82	0.70	0.79	0.88	0.83	0.76	0.81	0.82	
Phosphatidylcholine (C34:1)	0.83	0.75	0.78	0.82	0.82	0.74	0.91	0.81	
Phosphatidylcholine (C34:3)	1.02	1.18	1.17	1.09	1.15	1.04	1.04	1.23	
Phosphatidylcholine (C36:1)	0.86	0.88	0.73	0.84	0.79	0.90	0.96	0.76	
Phosphatidylcholine (C36:2)	0.66	0.71	0.62	0.69	0.71	0.65	0.86	0.78	
Phosphatidylcholine (C36:3)	0.90	1.03	0.84	0.96	0.97	0.89	0.93	0.82	
Phosphatidylcholine (C36:4)	1.00	0.93	0.81	0.98	0.95	0.94	0.98	0.66	

Phosphatidylcholine (C36:5)	0.78	0.81	0.63	0.76	0.72	0.75	0.92	0.71	
Phosphatidylethanolamine (C36:3)	0.90	0.87	0.87	0.90	0.87	0.84	0.82	0.86	
Phosphatidylethanolamine (C38:3)	0.72	0.70	0.64	0.75	0.68	0.66	0.92	1.05	
Phosphatidylethanolamine (C38:5)	0.90	1.00	0.86	0.98	0.87	0.90	0.89	0.82	
Phosphatidylethanolamine (C38:6)	0.92	0.91	0.95	1.01	0.98	0.92	0.96	0.91	
Phosphatidylethanolamine (C40:7)	0.79	0.81	0.75	0.88	0.82	0.88	0.92	0.68	
Lysophosphatidylcholine (C14:0)	2.27	1.82	2.09	1.97	1.85	1.87	1.51	1.75	Lysoglycerophospholipids
Lysophosphatidylcholine (C16:0)	1.86	1.92	1.98	2.08	1.93	2.14	1.26	1.17	
Lysophosphatidylcholine (C16:1)	1.95	2.13	2.01	2.17	1.86	1.72	1.06	1.22	
Lysophosphatidylcholine (C20:4)	1.22	1.67	1.47	1.90	1.86	1.44	1.28	0.94	
Lysophosphatidylcholine (C24:0)	1.01	1.18	1.11	1.20	1.21	1.29	1.50	1.25	
Lysophosphatidylcholine (C24:1)	1.10	1.03	1.13	1.26	1.10	1.12	1.20	1.00	
Lysophosphatidylethanolamine (C16:0)	2.47	2.37	2.23	2.12	2.10	2.19	1.18	1.67	
Lysophosphatidylethanolamine (C18:0)	2.21	2.07	1.76	2.07	1.77	2.04	1.26	1.69	
Lysophosphatidylethanolamine (C18:1)	2.45	2.31	1.97	2.19	2.11	2.16	1.12	1.09	
Ceramide (d18:1.C20:0)	0.63	0.66	0.56	0.62	0.64	0.59	0.66	0.74	Sphingolipids
Ceramide (d18:1.C24:1)	0.80	0.85	0.68	0.66	0.91	0.98	0.92	0.72	
Ceramide (d18:1.C24:2)	0.83	1.08	1.11	0.89	0.90	0.91	0.71	0.68	
Ceramide (d18:2.C24:1)	0.56	0.62	0.68	0.68	0.89	0.63	0.97	0.64	
Sphingomyelin (d32:2)	0.49	0.71	0.69	0.75	0.59	0.78	0.79	0.65	
Sphingomyelin (d33:1)	1.19	1.34	1.15	1.32	1.18	1.20	1.13	0.93	
Sphingomyelin (d34:0)	0.96	1.09	1.25	1.15	1.11	1.11	1.04	1.12	
Sphingomyelin (d35:1)	0.86	0.95	0.86	0.88	0.85	0.92	0.99	0.85	
Sphingomyelin (d36:1)	0.86	0.96	0.86	0.90	0.82	0.84	0.90	0.84	
Sphingomyelin (d36:2)	0.73	0.90	0.90	0.85	0.87	0.88	0.97	0.76	
Sphingomyelin (d37:1)	0.70	0.80	0.79	0.80	0.76	0.80	0.81	0.89	
Sphingomyelin (d38:1)	0.88	0.84	0.85	0.87	0.87	0.91	0.84	0.89	
Sphingomyelin (d38:2)	0.61	0.66	0.64	0.67	0.71	0.66	0.74	0.83	
Sphingomyelin (d39:1)	0.77	0.77	0.83	0.87	0.81	0.86	0.94	1.01	
Sphingomyelin (d40:2)	0.73	0.83	0.81	0.87	0.78	0.84	0.81	0.87	
Sphingomyelin (d41:2)	0.78	0.79	0.82	0.88	0.77	0.80	0.79	0.90	
Sphingomyelin (d42:2)	0.86	0.95	0.81	0.98	0.89	0.91	0.79	0.85	
Phosphocholine	0.78	0.69	0.56	0.69	0.63	0.63	0.91	0.79	Miscellaneous lipids

Suppl Fig. 10 Metabolome changes induced by liver enzyme inducers. *Heatmap of common statistically significantly (p<0.05) altered metabolites after Pendimethalin, Aroclor and β-naphthoflavone treatment. Red represents significantly upregulated metabolites and blue represents significantly downregulated metabolites as compared to controls. C3 for β-naphthoflavone and Pendimethalin and C2 for Aroclor (equivalent to EC15ATP) was used for the analysis (n=6 per condition).*

Metabolite	Aroclor 1254	ß-Naphthoflavone	Pendimethalin	Metabolite class
Proline	0.54	0.64	0.89	Amino acids
Tyrosine	1.13	1.14	1.14	
N-Acetylserine	1.07	1.03	1.47	Amino acids related
Cysteinylglycine	1.52	3.16	1.14	
myo-Inositol-2-phosphate	0.83	0.65	0.76	Carbohydrates and related
N-Acetylglucosamine	0.92	0.79	0.93	
Carnitine	0.68	0.88	0.76	Energy metabolism and related
O-Acetylcarnitine	0.81	0.87	1.02	
Propionylcarnitine	0.75	1.03	0.65	
Hexanoylcarnitine	0.68	1.02	0.69	
Pantothenic acid	0.76	0.96	0.78	Vitamins. cofactors and related
Flavin adenine dinucleotide (FAD)	1.11	1.63	1.14	

Glutathione (GSH)	1.16	2.59	1.12	
Triacylglycerol (C32:1.C16:1)	0.46	0.26	0.58	Acylglycerols
Triacylglycerol (C30:0.C18:1)	0.72	0.36	0.81	
Triacylglycerol (C32:0.C16:1)	0.58	0.41	0.66	
Triacylglycerol (C34:1.C16:0)	0.70	0.39	0.79	
Triacylglycerol (C34:2.C18:1)	0.49	0.24	0.44	
Triacylglycerol (C34:2.C18:0)	0.67	0.40	0.87	
Triacylglycerol (C34:1.C18:1)	0.82	0.47	0.71	
Triacylglycerol (C34:0.C18:1)	0.91	0.47	0.96	
Triacylglycerol (C36:3.C18:1)	0.65	0.38	0.78	
Triacylglycerol (C36:2.C18:1)	0.67	0.41	0.49	
Triacylglycerol (C36:1.C18:2)	0.82	0.46	0.83	
Triacylglycerol (C36:1.C18:1)	0.70	0.35	0.65	
Triacylglycerol (C36:1.C18:0)	0.78	0.46	0.86	
Phosphatidylethanolamine (C32:0)	2.54	1.63	3.04	Glycerophospholipids
Phosphatidylethanolamine (C34:2)	0.76	0.93	0.68	
Phosphatidylethanolamine (C34:1)	1.36	1.38	1.48	
Phosphatidylethanolamine (C34:0)	2.09	1.79	2.78	
Phosphatidylethanolamine (C36:3)	0.83	0.84	0.82	
Phosphatidylethanolamine (C36:1)	1.65	1.81	1.46	
Phosphatidylethanolamine (C36:0)	2.75	2.10	3.98	
Phosphatidylethanolamine (C38:5)	0.84	0.93	0.79	
Phosphatidylethanolamine (C40:7)	0.84	0.79	0.57	
Phosphatidylcholine (C32:0)	2.06	1.16	1.93	
Phosphatidylcholine (C34:3)	0.71	0.60	0.79	
Phosphatidylcholine (C34:2)	0.74	0.77	0.64	
Phosphatidylcholine (C34:0)	1.79	1.39	1.83	
Phosphatidylcholine (C36:4)	1.27	1.08	1.10	
Phosphatidylcholine (C36:3)	0.81	0.87	0.73	
Phosphatidylcholine (C36:2)	0.66	0.80	0.52	
Phosphatidylcholine (C36:1)	1.20	1.21	0.93	
Phosphatidylcholine (C36:0)	2.17	1.62	2.49	
Phosphatidylcholine (C38:6)	1.31	1.09	1.15	
Choline plasmalogen (C36:5)	0.86	0.84	0.68	
Choline plasmalogen (C36:4)	0.78	0.82	0.63	
Lysophosphatidylethanolamine (C16:0)	1.12	1.77	1.81	Lysoglycerophospholipids
Lysophosphatidylethanolamine (C18:0)	1.26	2.25	2.07	
Lysophosphatidylcholine (C14:0)	1.20	1.48	2.28	
Lysophosphatidylcholine (C16:0)	1.50	2.15	2.37	
Lysophosphatidylcholine (C20:4)	1.34	1.68	2.32	
Lysophosphatidylcholine (C20:1)	1.05	2.23	1.47	
Lysophosphatidylcholine (C20:0)	1.67	2.34	2.37	

Lysophosphatidylcholine (C22:0)	1.76	1.99	3.44	
Lysophosphatidylcholine (C24:1)	2.03	2.34	3.59	
Lysophosphatidylcholine (C24:0)	1.92	2.67	4.49	
Sphingomyelin (d32:1)	0.94	0.84	0.92	Sphingolipids
Sphingomyelin (d34:2)	1.22	1.05	1.11	
Sphingomyelin (d35:1)	1.17	1.06	1.07	
Sphingomyelin (d38:2)	0.85	0.94	0.76	
Sphingomyelin (d40:2)	0.98	0.77	0.93	
Sphingomyelin (d41:2)	0.88	0.85	0.79	
Sphingomyelin (d42:2)	0.84	0.74	0.73	
Sphingomyelin (d34:2)	1.35	1.07	1.27	
Ceramide (d18:2.C16:0)	2.15	1.48	2.06	
Ceramide (d18:1.C20:0)	0.97	0.73	0.81	
Ceramide (d18:1.C22:1)	0.75	0.65	0.43	
Ceramide (d16:1.C24:0)	1.58	1.20	1.35	
Ceramide (d18:2.C23:0)	1.29	1.56	1.14	
Ceramide (d17:1.C24:0)	1.42	1.24	1.40	
Ceramide (d18:2.C24:1)	1.18	1.23	1.17	
Ceramide (d18:2.C24:0)	1.91	1.41	1.87	
Ceramide (d18:1.C24:1)	0.75	0.64	0.54	
Isopentenyl pyrophosphate (IPP)	1.37	2.43	2.79	Cholesterol and related

Suppl Fig. 11 Metabolome changes induced by a liver enzyme inhibitor. *Heatmap of statistically significantly (p<0.05) altered metabolites after Ketoconazole treatment. Red represents significantly upregulated metabolites and blue represents significantly downregulated metabolites as compared to controls. C3 (equivalent to EC15ATP) was used for the analysis (n=6 per condition).*

**Metabolite**	**Ketoconazole**	**Metabolite class**
Threonine	0.85	Amino acids
Proline	0.79	
Glutamate	0.92	
Taurine	1.32	
Pipecolic acid	0.86	Amino acids related
N-Acetylserine	0.84	
N-Acetylaspartate	0.69	
S-Adenosylhomocysteine	0.69	
5-Hydroxytryptophan	0.87	
myo-Inositol-2-phosphate	0.50	Carbohydrates and related
Tetradecanoylcarnitine	2.20	Energy metabolism and related
Hexadecenoylcarnitine	3.61	
Hexadecanoylcarnitine	1.97	
Octadecenoylcarnitine	3.35	
Carnitine	0.77	
2'-Deoxycytidine	0.76	Nucleobases and related

Coenzyme Q10	0.65	Vitamins. cofactors and related
Pyridoxal	0.82	
Pantothenic acid	0.85	
Glutathione (GSH)	0.90	
Triacylglycerol (C30:0.C18:1)	0.61	Acylglycerols
Triacylglycerol (C32:0.C16:1)	0.51	
Triacylglycerol (C32:0.C16:0)	0.23	
Triacylglycerol (C34:1.C16:0)	0.44	
Triacylglycerol (C34:0.C16:0)	0.16	
Triacylglycerol (C34:0.C17:0)	0.34	
Triacylglycerol (C34:1.C18:3)	1.33	
Triacylglycerol (C36:4.C16:0)	1.15	
Triacylglycerol (C34:2.C18:1)	1.31	
Triacylglycerol (C34:2.C18:0)	0.68	
Triacylglycerol (C34:1.C18:1)	0.83	
Triacylglycerol (C34:0.C18:1)	0.31	
Triacylglycerol (C34:0.C18:0)	0.14	
Triacylglycerol (C36:3.C18:2)	2.03	
Triacylglycerol (C36:4.C18:0)	1.19	
Triacylglycerol (C36:3.C18:1)	1.57	
Triacylglycerol (C36:2.C18:1)	1.14	
Triacylglycerol (C36:1.C18:1)	0.61	
Triacylglycerol (C36:1.C18:0)	0.27	
Phosphatidylethanolamine (C32:0)	0.86	Glycerophospholipids
Phosphatidylethanolamine (C34:1)	0.82	
Phosphatidylethanolamine (C34:0)	0.73	
Phosphatidylethanolamine (C36:4)	0.96	
Phosphatidylethanolamine (C36:2)	0.93	
Phosphatidylethanolamine (C36:1)	0.78	
Phosphatidylethanolamine (C36:0)	0.70	
Phosphatidylethanolamine (C38:6)	0.86	
Phosphatidylethanolamine (C38:5)	1.10	
Phosphatidylethanolamine (C38:4)	1.10	
Phosphatidylethanolamine (C38:3)	0.90	
Phosphatidylethanolamine (C40:7)	1.09	
Phosphatidylcholine (C32:0)	0.81	
Phosphatidylcholine (C34:3)	1.19	
Phosphatidylcholine (C34:2)	0.89	
Phosphatidylcholine (C34:1)	0.79	
Phosphatidylcholine (C34:0)	0.81	
Phosphatidylcholine (C36:5)	0.66	
Phosphatidylcholine (C36:4)	0.67	
Phosphatidylcholine (C36:3)	0.89	
Phosphatidylcholine (C36:2)	0.64	
Phosphatidylcholine (C36:1)	0.77	
Phosphatidylcholine (C38:6)	0.74	

Phosphatidylcholine (C38:4)	1.06	
Phosphatidylcholine (C40:8)	1.11	
Phosphatidylcholine (C40:7)	1.16	
Phosphatidylcholine (C40:6)	1.10	
Choline plasmalogen (C36:5)	1.44	
Choline plasmalogen (C36:4)	1.23	
Lysophosphatidylethanolamine (C16:0)	0.82	Lysoglycerophospholipids
Lysophosphatidylethanolamine (C18:0)	1.22	
Lysophosphatidylethanolamine (C20:4)	4.71	
Lysophosphatidylethanolamine (C22:6)	4.79	
Lysophosphatidylcholine (C14:0)	1.21	
Lysophosphatidylcholine (C16:1)	2.67	
Lysophosphatidylcholine (C16:0)	1.96	
Lysophosphatidylcholine (C20:4)	6.43	
Lysophosphatidylcholine (C20:1)	1.07	
Lysophosphatidylcholine (C20:0)	1.34	
Lysophosphatidylcholine (C22:0)	1.53	
Lysophosphatidylcholine (C24:1)	2.21	
Sphingomyelin (d32:2)	1.28	Sphingolipids
Sphingomyelin (d32:1)	1.49	
Sphingomyelin (d33:1)	1.18	
Sphingomyelin (d34:2)	1.31	
Sphingomyelin (d34:1)	1.40	
Sphingomyelin (d34:0)	2.87	
Sphingomyelin (d35:2)	1.71	
Sphingomyelin (d35:1)	1.86	
Sphingomyelin (d36:3)	1.48	
Sphingomyelin (d36:2)	1.45	
Sphingomyelin (d36:1)	1.62	
Sphingomyelin (d37:1)	1.49	
Sphingomyelin (d38:2)	1.21	
Sphingomyelin (d38:1)	1.42	
Sphingomyelin (d39:1)	1.79	
Sphingomyelin (d40:2)	1.67	
Sphingomyelin (d40:1)	1.85	
Sphingomyelin (d41:2)	1.88	
Sphingomyelin (d41:1)	1.75	
Sphingomyelin (d42:2)	1.83	
Sphingomyelin (d42:1)	1.54	
Sphingomyelin (d34:1)	1.16	
Ceramide (d18:2.C16:0)	0.55	
Ceramide (d18:1.C16:0)	0.74	
Ceramide (d18:2.C18:0)	0.38	
Ceramide (d18:1.C18:0)	0.58	
Ceramide (d18:1.C20:0)	0.38	
Ceramide (d18:1.C21:0)	0.64	

Ceramide (d18:2.C22:0)	0.39	
Ceramide (d16:1.C24:0)	0.48	
Ceramide (d18:1.C22:0)	0.54	
Ceramide (d18:2.C23:0)	0.49	
Ceramide (d18:1.C23:0)	0.50	
Ceramide (d17:1.C24:0)	0.40	
Ceramide (d18:2.C24:2)	0.36	
Ceramide (d18:2.C24:1)	0.66	
Ceramide (d18:2.C24:0)	0.48	
Ceramide (d18:1.C24:1)	0.70	
Ceramide (d18:1.C24:0)	0.60	
Cholesterylester (C20:2)	0.58	Cholesterol and related
Isopentenyl pyrophosphate (IPP)	0.50	
Phosphocholine	0.82	Miscellaneous lipids
